# Adherence clubs and decentralized medication delivery to support patient retention and sustained viral suppression in care: Results from a cluster-randomized evaluation of differentiated ART delivery models in South Africa

**DOI:** 10.1371/journal.pmed.1002874

**Published:** 2019-07-23

**Authors:** Matthew P. Fox, Sophie Pascoe, Amy N. Huber, Joshua Murphy, Mokgadi Phokojoe, Marelize Gorgens, Sydney Rosen, David Wilson, Yogan Pillay, Nicole Fraser-Hurt

**Affiliations:** 1 Department of Global Health, Boston University School of Public Health, Boston, Massachusetts, United States of America; 2 Department of Epidemiology, Boston University School of Public Health, Boston, Massachusetts, United States of America; 3 Health Economics and Epidemiology Research Office, Department of Internal Medicine, School of Clinical Medicine, Faculty of Health Sciences, University of the Witwatersrand, Johannesburg, South Africa; 4 National Department of Health, Pretoria, South Africa; 5 The World Bank Group, Washington DC, United States of America; University of Southampton, UNITED KINGDOM

## Abstract

**Background:**

Differentiated antiretroviral therapy (ART) delivery models, in which patients are provided with care relevant to their current status (e.g., newly initiating, stable on treatment, or unstable on treatment) has become an essential part of patient-centered health systems. In 2015, the South African government implemented Chronic Disease Adherence Guidelines (AGLs), which involved five interventions: Fast Track Initiation Counseling for newly initiating patients, Enhanced Adherence Counseling for patients with an unsuppressed viral load, Early Tracing of patients who miss visits, and Adherence Clubs (ACs) and Decentralized Medication Delivery (DMD) for stable patients. We evaluated two of these interventions in 24 South African facilities: ACs, in which patients meet in groups outside usual clinic procedures and receive medication; and DMD, in which patients pick up their medication outside usual pharmacy queues.

**Methods and findings:**

We compared those participating in ACs or receiving DMD at intervention sites to those eligible for ACs or DMD at control sites. Outcomes were retention and sustained viral suppression (<400 copies/mL) 12 months after AC or DMD enrollment (or comparable time for controls). 12 facilities were randomly allocated to intervention and 12 to control arms in four provinces (Gauteng, North West, Limpopo, and KwaZulu Natal). We calculated adjusted risk differences (aRDs) with cluster adjustment using generalized estimating equations (GEEs) using difference in differences (DiD) with patients eligible for ACs/DMD prior to implementation (Jan 1, 2015) for comparison. For DMD, randomization was not preserved, and the analysis was treated as observational. For ACs, 275 intervention and 294 control patients were enrolled; 72% of patients were female, 61% were aged 30–49 years, and median CD4 count at ART initiation was 268 cells/μL. AC patients had higher 1-year retention (89.5% versus 81.6%, aRD: 8.3%; 95% CI: 1.1% to 15.6%) and comparable sustained 1-year viral suppression (<400 copies/mL any time ≤ 18 months) (80.0% versus 79.6%, aRD: 3.8%; 95% CI: −6.9% to 14.4%). Retention associations were apparently stronger for men than women (men RD: 13.1%, 95% CI: 0.3% to 23.5%; women RD: 6.0%, 95% CI: −0.9% to 12.9%). For DMD, 232 intervention and 346 control patients were enrolled; 71% of patients were female, 65% were aged 30–49 years, and median CD4 count at ART initiation was 270 cells/μL. DMD patients had apparently lower retention (81.5% versus 87.2%, aRD: −5.9%; 95% CI: −12.5% to 0.8%) and comparable viral suppression versus standard of care (77.2% versus 74.3%, aRD: −1.0%; 95% CI: −12.2% to 10.1%), though in both cases, our findings were imprecise. We also noted apparently increased viral suppression among men (RD: 11.1%; 95% CI: −3.4% to 25.5%). The main study limitations were missing data and lack of randomization in the DMD analysis.

**Conclusions:**

In this study, we found comparable DMD outcomes versus standard of care at facilities, a benefit for retention of patients in care with ACs, and apparent benefits in terms of retention (for AC patients) and sustained viral suppression (for DMD patients) among men. This suggests the importance of alternative service delivery models for men and of community-based strategies to decongest primary healthcare facilities. Because these strategies also reduce patient inconvenience and decongest clinics, comparable outcomes are a potential success. The cost of all five AGL interventions and possible effects on reducing clinic congestion should be investigated.

**Clinical Trial registration:**

NCT02536768.

## Abstract 2

Matthew Fox and colleagues report on outcomes of two tailored service delivery models for HIV therapy in South Africa.

## Author summary

**Why was this study done?:**

- Antiretroviral therapy (ART) has brought enormous benefits, enabling HIV patients to live longer and healthier lives. However, many patients struggle to adhere to this lifelong treatment, and their retention in care is suboptimal.
- To address this, South Africa’s National Department of Health has introduced National Adherence Guidelines for Chronic Diseases that recommend key interventions suitable for new patients, clinically stable patients, patients with advanced HIV disease or on a failing ART regimen, and lost-to-follow-up patients.
- The early implementation of these guidelines from 2015 was evaluated to understand the effectiveness of five interventions and learn about improvements for the guidelines’ rollout.
- This paper provides the results on adherence clubs and decentralized medication delivery, two interventions for efficient medication pick-up by clinically stable patients.

**What did the researchers do and find?:**

- We used a cluster-randomized evaluation design for adherence clubs and an observational study for decentralized medication delivery across 12 intervention and 12 control health facilities in 4 provinces.
- The 275 adherence club patients had improved retention in care at one year compared to the 294 controls, especially men, and comparable viral suppression within 18 months.
- The 232 patients receiving decentralized medication delivery had apparently lower retention in care than the 346 controls (possibly due to medication pick-up data not reaching clinics), and viral suppression was comparable overall, but men on decentralized medication delivery had apparently increased viral suppression.

**What do these findings mean?:**

- This programmatic evaluation with a comparison group provides the National Department of Health with many lessons for the national rollout of the guidelines and the effect one can expect from such alternative medication pick-up services.
- The control facilities were already offering some adherence and retention services, and the observed benefits of the guideline interventions are therefore additional.
- The comparable outcomes for decentralized medication delivery against standard of care, the retention benefit by adherence clubs, and the apparent increased effects among men suggest that these interventions can play an important role in South Africa’s ART program.
- Because the interventions also decentralize ART and reduce patients’ inconvenience, comparable outcomes are a potential success, and further work should investigate the cost–benefit relation and possible effects on decongesting clinics.

## Introduction

The benefits of the rollout of antiretroviral therapy (ART) programs in resource-limited settings have been massive, including increased survival [1,2], reduced morbidity [3–6], and potential reductions in transmission [7,8]. Now that HIV treatment programs have had time to mature, attention has turned to the challenges of keeping patients adherent to lifelong therapy. Ample evidence from studies that have reviewed evidence from all over sub-Saharan Africa has shown that retention in HIV care is suboptimal. Current evidence suggests that 5-year retention in sub-Saharan Africa is close to 60% [9–14].

One way to attempt to improve retention is by reducing the burden on the patient of seeking care. Differentiated ART delivery models, in which patients with different needs receive tailored care [15–18], has been proposed as a solution. Under differentiated ART delivery, patients who are clinically stable on treatment and have demonstrated good adherence can be offered a repeat prescription collection strategy that allows them to pick up their medication in a less time-consuming manner than general clinic pharmacy queues as part of a package of services, including counseling and peer support. Two such approaches are Adherence Clubs (ACs), in which patients meet in a small groups outside the usual clinic queues, pick up their prepacked medication, and discuss adherence, and Decentralized Medication Delivery (DMD), in which patients pick up their medication at a pick-up point (PuP) away from the clinic such as a private pharmacy or church. For both strategies, medication may be prepacked by the clinic, a government district pharmacist, or a private provider. Such approaches may provide an incentive for already clinically stable patients to continue to adhere because the time burden for medication collection is reduced. They may also reduce the burden on the clinic because these patients require fewer clinic visits. While the literature does show some benefit of repeat prescription strategies along with a package of services in differentiated ART delivery, studies have largely focused on the effectiveness of ACs, with most data coming from observational studies [19–22]. Further, most studies were evaluations of programs that did not mimic routine conditions but rather evaluated pilot programs with enhanced or nongovernmental organization (NGO) support. Thus, while evidence is beginning to emerge on the effectiveness of differentiated ART delivery models, to date, there is not enough evidence to conclude the approaches are effective.

South Africa, which has the largest HIV treatment program in the world [23], has been grappling with retention and poor adherence [24,25] and, in response, developed a comprehensive strategy to tackle these issues. The approach was developed in 2014 by the National Department of Health (NDOH) and is described in the “National Adherence Guidelines for Chronic Diseases” [26] (AGLs), which call for a “minimum package” of interventions to mitigate the problem by identifying patients who require more intense care (patients with unsuppressed viral loads and those lost from care) and those who require less intense care (patients who have demonstrated an ability to achieve and maintain viral suppression). The AGL minimum package of interventions contains eight interventions focused on education and counseling, repeat prescription strategies, patient tracing, and integrated care, detailed in a set of guidelines [26] and standard operating procedures [27].

In order to develop an evidence base behind differentiated ART delivery models and to inform improvement of the rollout of the interventions, South Africa’s NDOH piloted rollout of the interventions in 12 primary healthcare clinics (PHCs) from 2015. We evaluated five of the interventions and present here an evaluation of two strategies, ACs and DMD, for clinically stable patients designed to reduce patient burden, which would in turn help clinicians and staff to provide increased support to clients with advanced HIV disease and for those on a failing ART regimen. The evaluation design has been reported previously [28,29].

## Methods

### Study design

The study used an unblinded cluster-randomized evaluation design for ACs and an observational study for DMD. For DMD, while the study was initially designed to have sites randomized to DMD roll out, changes in clinic mandates meant that some clinics began using DMD in control sites and not all intervention sites were able to begin implementing DMD. The full protocol can be seen in S1 Text. The study was conducted in 24 health facilities (12 intervention, 12 control sites) in Gauteng, KwaZulu Natal, Limpopo, and North West provinces. Details on the sites and viral suppression by site is provided in S1 Table. Matched clinic pairs were randomized 1:1 (by computer) by the NDOH to intervention and control with a single randomization for all interventions. Intervention sites implemented the AGL interventions, and control sites continued to provide standard of care. Standard of care for clinically stable patients would include a visit usually every other month. Some counseling may have been given during visits, and some facilities may have offered support groups or group counseling sessions in the waiting areas. In addition, four control sites implemented AC-like interventions as part of standard of care. All 24 sites were PHCs that were 1) high volume (total on ART > 1,000 as reported by sites, which was not always accurate); 2) not a National Health Insurance pilot site; 3) generating computerized TIER.Net data; and 4) not participating in other adherence-related studies. Sites were matched on district, total on ART, proportion virally suppressed, setting (rural/urban/formal/informal), and location.

### Interventions

ACs comprise clinically stable ART patients who meet at facilities or community locations in groups of up to 30 every 2 to 3 months to receive group counseling, have a brief symptom screen, and receive prepacked medications. Clubs are managed by lay staff and nurses at the facility with support from community health workers. The goal is to keep patients engaged and adherent by providing social support and facilitating medication delivery and treatment monitoring while reducing patient burden at the clinic because these patients only require 6-monthly clinic visits and assessments for blood tests and rescripting. While each of the components of the ACs (prepacked medications, social support, adherence counseling, etc.) are currently considered important parts of the intervention, to date, none of the components have been tested alone to determine which components are essential.

DMD comprises prepacking and distribution of medications to PuPs, which are at locations other than the clinic pharmacy. Patients only need to come to the clinic on a 6-monthly basis for a clinical exam and rescripting. The goal is to reduce the time and resource burden on the patient while also decongesting clinics. DMD was established using three models: 1) facility-led prepackaging of medicines delivered to external sites; 2) Centralized Chronic Medicine Dispensing and Distribution (CCMDD), in which private partners prepackaged medicines and delivered them to the facility, which then distributed packs to dedicated PuPs (such as an AC) or delivered direct to a pharmacy or other dedicated PuP; and 3) chronic dispensing unit, in which the district pharmacist prepackaged medication and delivered to facilities, PuPs, or ACs. For clarity, we note that patients enrolled in the DMD analyses could not be in an AC.

DOH staff at facilities started to be trained on these interventions in July 2015, and training continued until mid-2016. At the start of data collection, the intervention sites had been implementing these interventions for at least 1 month and at some sites up to 6 months. At intervention sites, clinically stable patients were supposed to be given a choice between any of the three repeat prescription collection strategies available: ACs, DMD, or spaced fast-lane appointments (which we did not evaluate), though in some cases, facilities did decide which intervention a patient would be offered. Patients could also choose to switch between strategies, but this was monitored in our cohorts, and very few switches were recorded. It was not possible, however, for a patient to be enrolled in more than one approach at the same time.

### Eligibility criteria

We included patients over 18 years old who were resident in the facility’s catchment area, had no documented plan to transfer facilities, and were neither pregnant nor eligible for prevention of mother-to-child transmission services. Patients had to be eligible for a repeat prescription collection strategy by 1) being on the same ART regimen for at least 12 months, 2) having had their most recent viral load in the past 3 months, and 3) having had two consecutive undetectable viral loads (<400 copies/mL).

### Enrollment

**Fig 1:**
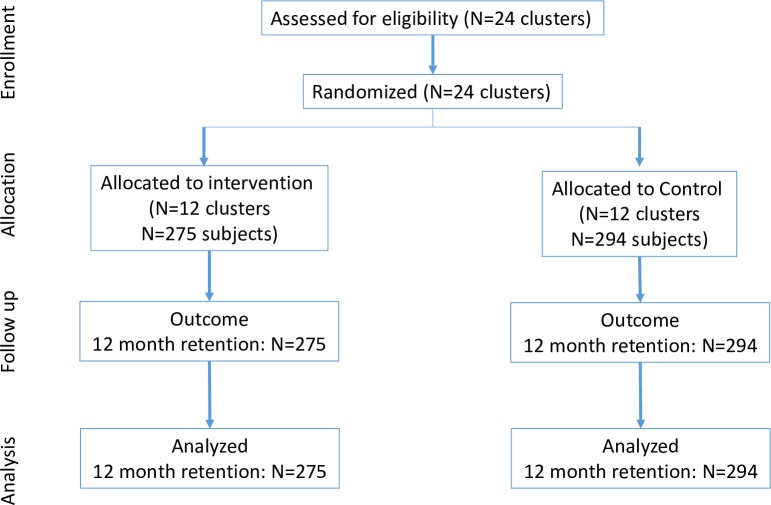
CONSORT flow chart for ACs. AC, Adherence Club.

**Fig 2:**
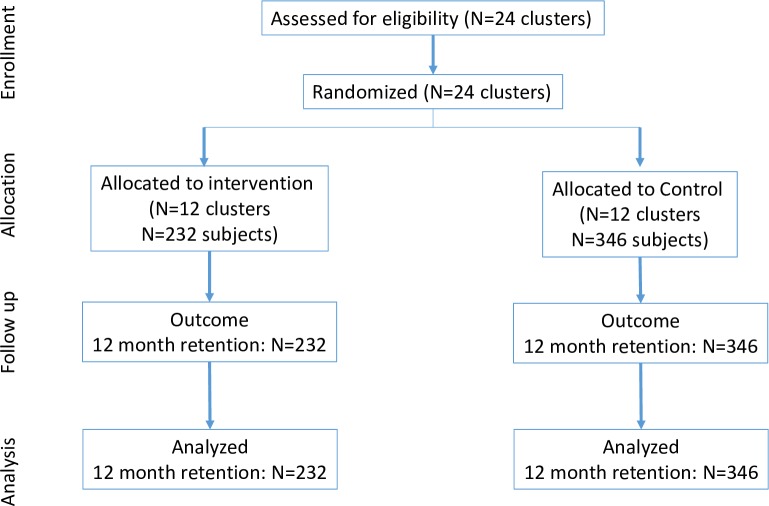
CONSORT flow chart for DMD. DMD, Decentralized Medication Delivery.

As the study was designed to assess retention-based outcomes, we did not interact with patients because this could impact outcomes. Instead, we received a waiver of consent and reviewed medical records to identify eligible patients. Follow-up started when patients became eligible to receive the intervention: the date of prescription visits for ACs/DMD for intervention patients and, for control patients, the clinic visit that occurred closest to the date that the medical record was reviewed. Follow-up start dates were between March 2016 and March 2017. At intervention sites, we included patients identified as actually having received the intervention (AC or DMD) in a clinic register or patient files. When more patients than required could be identified, we took a random sample of those eligible (for ACs, from all possible ACs). We then confirmed eligibility with clinic files. At control sites, we used clinic records to identify persons who would have been eligible for a repeat prescription strategy if they had been implemented at the facility and randomly allocated (using a computer) eligible patients to ACs or DMD to serve as controls (e.g., even random numbers were allocated to ACs, and odd random numbers were allocated to DMD). Again, we took a random sample of all those eligible. However, we note that because these participants were not offered the intervention, we could not identify which control subjects would have received the intervention if offered, and this could lead to some lack of comparability in our populations. Study flow figures for ACs and DMD are presented in Figs 1 and 2; the CONSORT checklist is presented in **S1 CONSORT Checklist**.

### Follow-up

We followed patients through routinely collected facility- and patient-level records. Follow-up data came from three sources: 1) clinic records (clinic registers, AC registers, and patient clinic files), 2) a national electronic patients database called TIER.Net, and 3) the National Health Laboratory Service (NHLS) database of all viral loads done in South Africa’s public sector [25,30]. Passive follow-up through medical record review continued for 18 months (to allow a 6-month window for 1-year outcomes to occur).

### Study outcomes

Our first long-term outcome was sustained viral suppression (<400 copies/mL) at 12 months after eligibility for ACs or DMD, indicated by any suppressed viral load from TIER.Net, NHLS, or paper files. We did not specify a window around 12 months for suppression, so we defined this post hoc based on any suppressed viral load within 2 to 18 months after eligibility for ACs or DMD to allow time for viral loads to be recorded. If there were discordant results within the follow-up period (one suppressed and one unsuppressed viral load result), the outcome was classified as suppressed. We note that we consider this as continued viral suppression because everyone in the study had to have a suppressed viral load at entry, even though in some cases (N = 18 AC and N = 20 DMD), patients did have both an unsuppressed and suppressed viral load in that time period. We also report viral suppression among those patients who had a viral load for comparison to the results including all patients.

Our second outcome was retention in care at 12 months after eligibility for ACs or DMD defined as 100% − % attrition, with attrition as the sum of reported deaths, loss to follow-up, and transfers. Loss to follow-up was defined based on clinic definitions—failure to attend the clinic within 90 days of a scheduled appointment. For retention, we used TIER.Net and file review to measure 12-month outcomes. The database was locked on May 23, 2018.

### Sample size and data analysis

We designed the study to have power = 80%, two-sided alpha = 0.05, and a coefficient of variation of 0.1 to account for clustering. We assumed 80% of patients would reach the primary outcome in the control arm. To detect a difference of 15%, we estimated we would need 24 subjects per clinic for each intervention (576 total).

We followed a similar analytic plan for each intervention and outcome. We compared arms on the proportion with the outcome and calculated crude risk differences and 95% CIs. Because we included those who actually received the intervention, our results should be considered as an “as treated” analysis. Next, we accounted for clustering using a linear regression generalized estimating equation (GEE) model of risk with an unstructured correlation matrix with site clustering. The model was then adjusted for differences in baseline covariates. Despite the cluster-randomized design, we anticipated differences between groups in the period prior to the intervention period. To account for this, we implemented a difference-in-differences (DiD) approach using data from a period prior to the intervention rollout (Jan 1, 2015 through Dec 31, 2015). Our model is

$$outcome_{ij}=\beta_{1}+\beta_{2}*period+\beta_{3}*intervention+\beta_{4}*period*intervention+Ѳ*X_{ij}+\mu_{ij},$$

where outcome_ij_ is a binary outcome for person i at site j, period is a dummy variable indicating the period (1 = intervention, 0 = preintervention), intervention is a dummy variable indicating randomization group (0 = control, 1 = intervention), and β_4_ is the effect of the intervention (i.e., difference between intervention and control groups in the intervention period minus differences in the preperiod). The model is further adjusted for a vector of covariates X_ij_ (which included sex, age, and CD4 count at ART initiation) and for clustering by clinic using GEEs. We also conducted subgroup analyses by age and sex, but these were not prespecified subgroup analyses and, as such, should be considered hypothesis generating. We note further that the decision to add in the DiD analysis was not in our initial protocol but added prior to data analysis, once we determined full randomization could not be maintained and we could not identify all control patients who would have received the interventions if offered.

### Ethical issues

The study was approved by the Human Research Ethics Committee (HREC) of the University of the Witwatersrand and the Boston University Institutional Review Board (IRB). Both approved use of routine clinic data for the evaluation and a waiver of consent. The trial is registered at clinicaltrials.gov (NCT02536768).

## Results

### ACs

Table 1 presents the baseline data, and the CONSORT figure is shown in Fig 1. Our sample was largely female (72%) and aged 30 to 49 years (61%). Median CD4 count at ART initiation was 268 cells/ml^3^. Arms were well balanced with respect to baseline demographics, but we saw some small imbalance in CD4 count at ART initiation (median 278 versus 256), and those in the intervention arm had been on treatment for substantially longer than the control arm.

**Table 1:** Baseline characteristics of the AC cohort by intervention and control status.

	AC Intervention		AC Control		AC Total	
	N = 275		N = 294		N = 569	
Characteristic	*n*	(%)	*n*	(%)	*n*	(%)
**Age (n = 569)**						
18–29	58	(21%)	61	(21%)	119	(21%)
30–39	100	(36%)	108	(37%)	208	(37%)
40–49	72	(26%)	68	(23%)	140	(25%)
50+	45	(16%)	57	(19%)	102	(18%)
**Gender (n = 569)**						
Female	206	(75%)	204	(69%)	410	(72%)
Male	69	(25%)	90	(31%)	159	(28%)
CD4 count (at ART initiation) (n = 249 control; 209 intervention)*	256 (148–355)		278 (168–406)		268 (157–379)	
**Viral load (copies/ml) (median, IQR) (n = 569)**	50 (20–124)		50 (20–124)		50 (20–124)	
**Log**_**10**_ **viral load (copies/ml) (median, IQR) (n = 569)**	1.70 (1.30–2.09)		1.70 (1.30–2.09)		1.70 (1.30–2.09)	
**Proportion below viral load lower limit of detection**						
** <125 copies/mL**	235	(85%)	250	(85%)	485	(85%)
** ≥125 copies/mL**	40	(15%)	44	(15%)	84	(15%)
**TB status (n = 569)**						
Current TB diagnosis	0	(0%)	1	(1%)	1	(1%)
No current TB diagnosis	275	(100%)	293	(99%)	568	(99%)
**Time on ART at enrollment (days) (median, IQR) (n = 569)**	839 (551–1,163)		577 (472–860)		714 (506–938)	

*We note that the AC cohort is not limited to those who were treatment naïve, so some patients could have been transfer-in patients without a baseline CD4 count. Others may have had a lost file and no record of the baseline CD4 count.

**Abbreviations:** AC, Adherence Club; ART, antiretroviral therapy; TB, tuberculosis.

### Viral suppression

**Fig 3:**
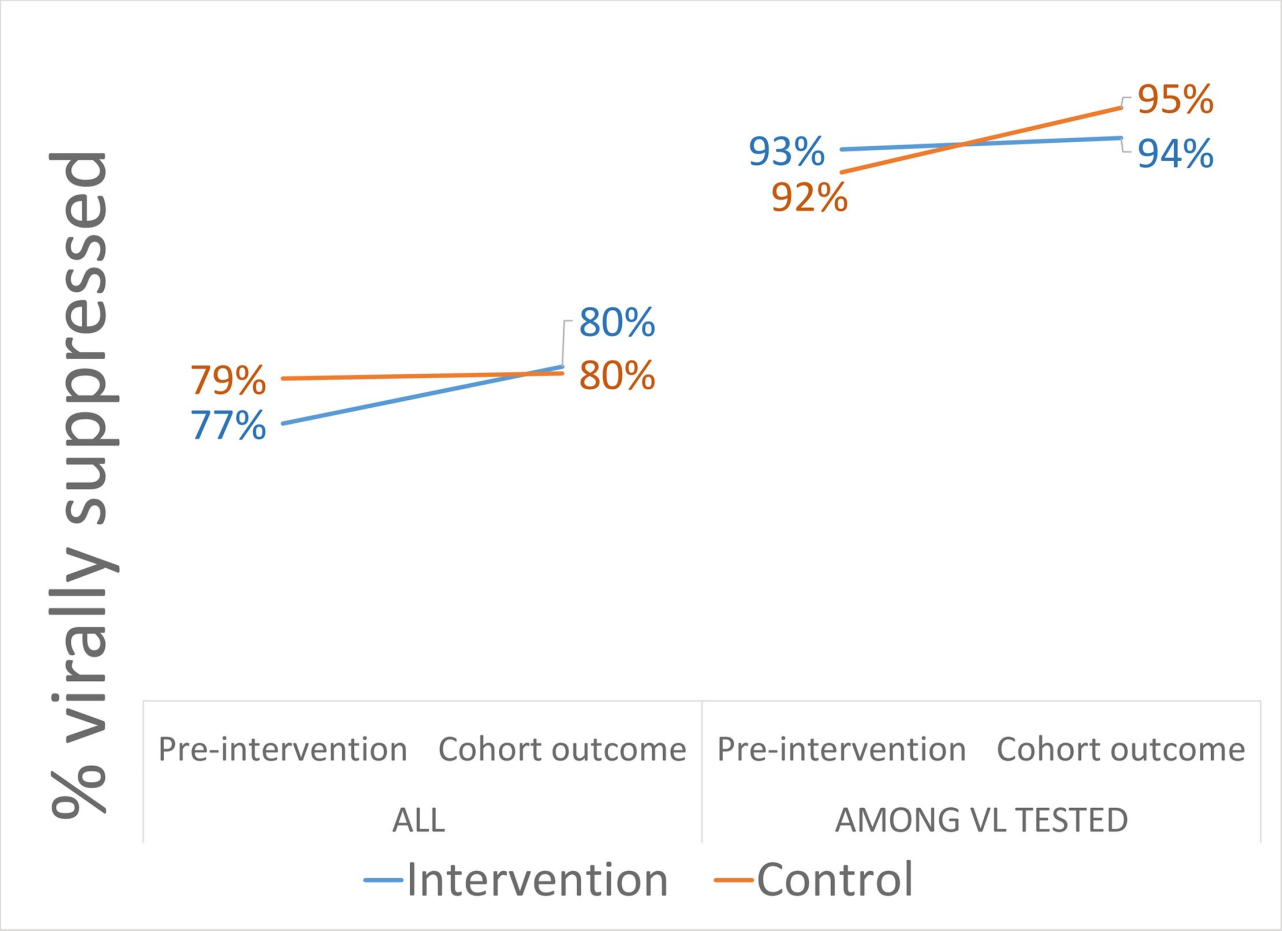
DiD proportions for viral suppression at 12 months (defined as within 2–18 months) among those eligible for ACs in the period prior to the interventions (preintervention) and among those enrolled (cohort outcome). AC, Adherence Club; DiD, difference in differences; VL, viral load.

About 84% of subjects had a repeat viral load within 18 months of club eligibility, but this did not differ by arm. Sustained viral suppression was high overall at about 80%, including those without a repeat viral load (suppression among those with a viral load was about 95%). This is expected given that those eligible for ACs are highly adherent already. In our crude analysis, ACs were associated with little change in sustained viral suppression (risk difference [RD]: 0.4%; 95% CI: −6.2% to 7.0%) (Table 2). We also saw higher sustained suppression in control sites (RD:−2.7%; 95% CI: −4.0% to −1.4%) prior to intervention rollout (S2 Table). When differences in sustained suppression prior to intervention rollout were combined with the enrolled cohort using DiD, we saw comparable suppression within 18 months (RD: 3.1%; 95% CI: −3.8% to 10.0%), and when adjusting for individual characteristics, CIs widened (RD: 3.8%; 95% CI: −6.9% to 14.4%) (full model in S3 Table). Overall, we saw evidence for comparable viral load outcomes between arms. We found little difference between intervention arms in sustained viral suppression results overall (Fig 1, left) and limited to those with a repeat viral load (Fig 3, right).

**Table 2:** Sustained viral suppression at 12 months (defined as within 2–18 months) for those eligible for ACs in the enrolled cohort and DiD analysis*.

Intervention						Control					
Facility	N	No VL	Suppressed	% Suppressed	% Suppressed with a VL	Facility	N	No VL	Suppressed	% Suppressed	% Suppressed with a VL
GP Site 1	23	2	21	91.3	100	GP Site 4	24	2	21	87.5	95.5
GP Site 2	28	2	22	78.6	84.6	GP Site 5	24	0	20	83.3	83.3
GP Site 3	8	2	5	62.5	83.3	GP Site 6	24	4	18	75.0	90.0
LP Site 1	24	4	20	83.3	100	LP Site 4	24	5	18	75.0	94.7
LP Site 2	24	3	18	75.0	85.7	LP Site 5	24	3	20	83.3	95.2
LP Site 3	24	2	21	87.5	95.5	LP Site 6	25	5	17	68.0	85.0
NW Site 1	24	2	21	87.5	95.5	NW Site 4	24	6	18	75.0	100
NW Site 2	24	2	22	91.7	100	NW Site 5	24	4	20	83.3	100
NW Site 3	24	6	18	75.0	100	NW Site 6	25	6	18	72.0	94.7
KZN Site 1	24	4	19	79.2	95.0	KZN Site 4	27	4	23	85.2	100
KZN Site 2	24	9	15	62.5	100	KZN Site 5	25	5	20	80.0	100
KZN Site 3	24	6	18	75.0	100	KZN Site 6	24	2	21	87.5	100
**Total**	275	44	220	80.0	95.2	**Total**	294	46	234	79.6	94.4
RD in % suppressed**	0.4% (−6.2% to 7.0%)										
RD in the preperiod**	−2.7% (−4.0% to −1.4%)										
DiD**	3.1% (−3.8% to 10.0%)										
DiD (cluster adjusted)***	3.1% (−7.9% to 14.1%)										
DiD (covariate adjusted and cluster adjusted)***	3.8% (−6.9% to 14.4%)										

*DiD analysis compares the enrolled cohort to all those who would have been eligible for ACs in the period prior to the rollout of the interventions (Jan 1, 2015 through Dec 31, 2015) (preperiod).

**Note that this is a crude analysis, with no adjustment for clustering or covariates as is done for the final model.

***Analyses are adjusted for clustering by site using a GEE with site-level clustering and an unstructured correlation matrix; note that sample size is smaller for the DiD covariate adjusted because those with missing data will drop out of the analysis.

**Abbreviations:** AC, Adherence Club; DiD, difference in differences; GEE, generalized estimating equation; GP, Gauteng Province; KZN, KwaZulu Natal; LP, Limpopo Province; NW, North West; RD, risk difference; VL, viral load.

### Retention

Retention at 12 months was high in both arms (85%), as expected given the targeted population. ACs were associated with a crude 7.8 (95% CI: 2.1% to 13.6%) percentage-point increase in retention (Table 3). Prior to intervention rollout, we saw no difference in retention between arms (RD: 0.4%; 95 CI: −0.4% to 1.2%) (S4 Table). Using DiD, we saw an increase in 12-month retention among those on ACs (RD: 7.4%; 95% CI: 2.9% to 11.9%) compared to controls and a similar result when controlling for clustering and individual characteristics (RD: 8.3%; 95% CI: 1.1% to 15.6%) (full model in S5 Table). Further, retention effects were apparently twice as large for men (RD: 13.1%; 95% CI: 0.3% to 23.5%) than women (RD: 6.0%; 95% CI: −0.9% to 12.9%), though estimates were imprecise. There seemed to be little benefit to those under 30, while we observed somewhat large benefits in some older age groups (S6 Table). This was true even though absolute values of retention increased with increasing age.

**Table 3:** Retention (alive and in care) at 12 months for those eligible for ACs in the enrolled cohort and DiD analysis*.

Intervention						Control					
Facility	N	Transfer	Died/LTF	Alive	% retained	Facility	N	Transfer	Died/LTF	Alive	% retained
GP Site 1	23	0	1	22	95.7	GP Site 4	24	0	5	19	79.2
GP Site 2	28	0	1	27	96.4	GP Site 5	24	0	1	23	95.8
GP Site 3	8	0	3	5	62.5	GP Site 6	24	1	3	20	83.3
LP Site 1	24	0	2	22	91.7	LP Site 4	24	2	2	20	83.3
LP Site 2	24	1	2	21	87.5	LP Site 5	24	1	1	22	91.7
LP Site 3	24	0	1	23	95.8	LP Site 6	25	0	1	24	96.0
NW Site 1	24	0	1	23	95.8	NW Site 4	24	4	6	14	58.3
NW Site 2	24	0	1	23	95.8	NW Site 5	24	1	6	17	70.8
NW Site 3	24	1	1	22	91.7	NW Site 6	25	0	5	20	80.0
KZN Site 1	24	2	1	21	87.5	KZN Site 4	27	0	5	22	81.5
KZN Site 2	24	1	7	16	66.7	KZN Site 5	25	2	4	19	76.0
KZN Site 3	24	0	3	21	87.5	KZN Site 6	24	0	4	20	83.3
**Total**	275	5	24	246	89.5	**Total**	294	11	43	240	81.6
RD**	7.8% (2.1% to 13.6%)										
RD in the preperiod**	0.4% (−0.4% to 1.2%)										
DiD**	7.4% (2.9% to 11.9%)										
DiD (cluster adjusted)***	7.4% (0.2% to 14.7%)										
DiD (covariate adjusted and cluster adjusted)***	8.3% (1.1% to 15.6%)										

*DiD analysis compares the enrolled cohort to all those who would have been eligible for ACs in the period prior to the rollout of the interventions (Jan 1, 2015 through Dec 31, 2015) (preperiod).

**Note that this is a crude analysis, with no adjustment for clustering or covariates as is done for the final model.

***Analyses are adjusted for clustering by site using a GEE with site-level clustering and an unstructured correlation matrix; note that sample size is smaller for the DiD covariate adjusted because those with missing data will drop out of the analysis.

**Abbreviations:** AC, Adherence Club; DiD, difference in differences; GEE, generalized estimating equation; GP, Gauteng Province; KZN, KwaZulu Natal; LP, Limpopo Province; LTF, Lost to follow-up; NW, North West; RD, risk difference.

### DMD results

The DMD cohort baseline characteristics were similar to the AC cohort, with roughly 65% between 30 and 49 years and about 70% female. Median CD4 count at ART initiation was 270 cells/ml^3^ at enrollment. Intervention and control arms were similar with respect to sex and age, but again, we found some small imbalances in CD4 (279 versus 256 cells/ml^3^) and log viral load at eligibility (log_10_ 1.62 versus 2.09 copies/ml) (Table 4, and the CONSORT figure is shown in Fig 3). As with ACs, those in the DMD intervention arm had been on treatment for substantially longer than the control arm. For DMD, because we were not able to preserve randomization, we included clinics actually implementing DMD in the intervention arm and those that were not in the control arm.

**Table 4:** Baseline characteristics of the DMD cohort by intervention and control status.

	DMD Intervention		DMD Control		DMD Total	
	N = 232		N = 346		N = 578	
Characteristic	*n*	(%)	*n*	(%)	*n*	(%)
**Age (n = 578)**						
18–29	38	(16%)	67	(19%)	105	(18%)
30–39	90	(39%)	116	(34%)	206	(36%)
40–49	70	(30%)	99	(29%)	169	(29%)
50+	34	(15%)	64	(18%)	98	(17%)
**Gender (n = 578)**						
Female	169	(73%)	239	(69%)	408	(71%)
Male	63	(27%)	107	(31%)	170	(29%)
**CD4 count (at ART initiation) (control n = 276, intervention n = 201)**	256 (137–349)		279 (142–386)		270 (142–365)	
Viral load (copies/ml) (median, IQR) (n = 576)**	124 (35–124)		42 (20–100)		50 (20–124)	
**Log**_**10**_ **viral load (copies/ml) (median, IQR) (n = 5,786)**	2.09 (1.54–2.09)		1.62 (1.30–2.22)		1.69 (1.30–2.09)	
**Proportion below viral load lower limit of detection**						
** <125 copies/mL**	204	(89%)	296	(86%)	500	(87%)
** ≥125 copies/mL**	26	(11%)	50	(14%)	76	(13%)
**TB status at study enrollment (n = 573)**						
Current TB diagnosis	1	(1%)	0	(0%)	1	(1%)
No current TB diagnosis	231	(99%)	341	(100%)	572	(99%)
**Time on ART at enrollment (days) (median, IQR) (n = 578)**	856 (592–1,028)		633 (454–884)		769 (491–935)	

**Note that 2 viral loads were not found.

**Abbreviations:** ART, antiretroviral therapy; DMD, Decentralized Medication Delivery; TB, tuberculosis.

### Viral suppression

**Fig 4:**
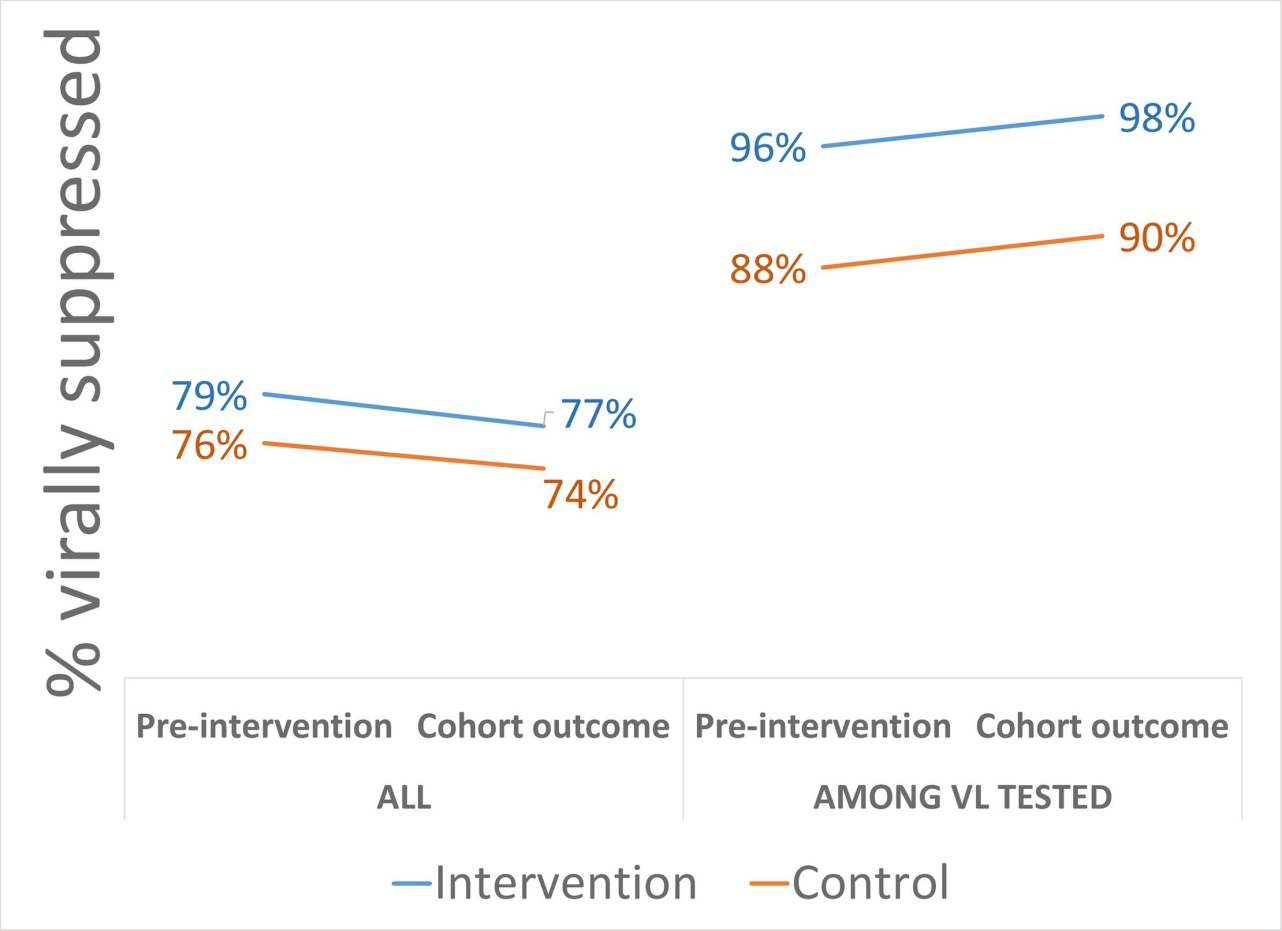
DiD in viral suppression at 12 months (defined as within 2–18 months) among those eligible for DMD in the period prior to the interventions (preintervention) and among those enrolled (cohort outcome). DiD, difference in differences; DMD, Decentralized Medication Delivery; VL, viral load.

We found that sustained suppression rates were only about 75% overall, but this was largely because of patients without a repeat viral load. Among those with a repeat viral load, suppression was about 95%. Comparing DMD implementation sites to those not implementing DMD, sustained suppression was similar (RD: 2.9%; 95% CI: −4.2% to 10.0%) (Table 5). Prior to intervention rollout, sustained suppression was slightly higher in implementation sites compared to control sites (RD: 3.3%; 95% CI: 2.0% to 4.6%) (S7 Table). Controlling for baseline differences, we found no difference in sustained suppression (RD: −0.5%; 95% CI: −7.5% to 6.6%). Results were largely unchanged when adjusting for clustering and individual characteristics (RD: −1.0%; 95% CI: −12.2% to 10.1%) (full model in S8 Table), though results were less precise. Further, while there was no benefit overall, there may be some among men (RD: 11.1%; 95% CI: −3.4% to 25.5%) (S9 Table). When limited to those with a viral load, viral suppression was sustained in 8% more of those patients in the DMD compared to the standard of care (from 90.2% to 98.3%) in the intervention period, though this disappeared using DiD with cluster adjustment (RD: −2.2%; 95% CI: −11.7% to 7.3%, data not presented in tables). Still, this suggests sustained suppression among those tested is higher in implementation sites, while repeat testing may be somewhat worse. Fig 4 presents the full results on the left and limited to those with a viral load on the right.

**Table 5:** Sustained viral suppression at 12 months (defined as within 2–18 months) for those eligible for DMD in the enrolled cohort and DiD analysis*.

DMD Implemented						DMD Not Implemented					
Facility	N	No VL	Suppressed	% Suppressed	% Suppressed with a VL	Facility	N	No VL	Suppressed	% Suppressed	% Suppressed with a VL
**GP Site 1**	8	1	7	87.5	100	**GP Site 2**	24	2	20	83.3	90.9
**Gp Site 4**	28	3	23	82.1	92.0	**GP Site 5**	24	5	16	66.7	84.2
**NW Site 1**	22	6	16	72.7	100	**GP Site 3**	26	4	13	50.0	59.1
**NW Site 2**	25	10	15	60.0	100	**GP Site 6**	24	2	20	83.3	90.9
**NW Site 5**	24	1	23	95.8	100	**LP Site 1**	24	2	22	91.7	100
**NW Site 3**	24	10	14	58.3	100	**LP Site 4**	26	0	24	92.3	92.3
**NW Site 6**	24	6	17	70.8	94.4	**LP Site 2**	24	6	16	66.7	88.9
**KZN Site 1**	24	5	19	79.2	100	**LP Site 5**	24	3	16	66.7	76.2
**KZN Site 2**	26	3	23	88.5	100	**LP Site 3**	24	12	12	50.0	100.0
**KZN Site 5**	27	5	22	81.5	100	**LP Site 6**	24	3	18	75.0	85.7
						**NW Site 4**	19	5	14	73.7	100
						**KZN Site 4**	27	8	19	70.4	100
						**KZN Site 3**	32	6	25	78.1	96.2
						**KZN Site 6**	26	3	22	84.6	95.7
**Total**	232	50	179	77.2	98.4	**Total**	346	61	257	74.3	90.2
RD in % suppressed overall**	2.9% (−4.2% to 10.0%)										
RD in the preperiod**	3.3% (2.0% to 4.6%)										
DiD**	−0.5% (−7.5% to 6.6%)										
DiD (cluster adjusted)***	−0.5% (−11.8% to 10.9%)										
DiD (covariate adjusted and cluster adjusted)***	−1.0% (−12.2% to 10.1%)										

*DiD analysis compares the enrolled cohort to all those who would have been eligible for DMD in the period prior to the rollout of the interventions (Jan 1, 2015 through Dec 31, 2015) (preperiod).

**Note that this is a crude analysis, with no adjustment for clustering or covariates as is done below for the final model.

***Analyses are adjusted for clustering by site using a GEE with site-level clustering and an unstructured correlation matrix; note that sample size is smaller for the DiD covariate adjusted because those with missing data will drop out of the analysis.

**Abbreviations:** DiD, difference in differences; DMD, Decentralized Medication Delivery; GEE, generalized estimating equation; GP, Gauteng Province; KZN, KwaZulu Natal; LP, Limpopo Province; NW, North West; RD, risk difference; VL, viral load.

### Retention

Retention was high overall (about 85%) with, apparently, somewhat lower retention in DMD than control sites (RD: −5.8%; 95% CI: −11.7% to 0.2%). Prior to intervention, we found nearly identical retention (RD 0.3%; 95% CI: −0.5% to 1.1%) (S10 Table). Adjusting for baseline differences using DiD, we found decreased retention at intervention sites (RD −6.0%; 95% CI: −10.6% to−1.1%) (Table 6) that changed little when adjusting for baseline differences between arms (RD −5.9%; 95% CI: −12.5% to 0.8%) (full final model in S11 Table). As with suppression the difference in retention, if real, appears to be largely among men (S12 Table).

**Table 6:** Retention (alive and in care) at 12 months for those eligible for DMD in the enrolled cohort and DiD analysis*.

DMD Implemented						DMD Not Implemented					
Facility	N	Transfer	Died/LTF	Alive	% retained	Facility	N	Transfer	Died/LTF	Alive	% retained
**GP Site 1**	8	0	0	8	100	**GP Site 2**	24	0	2	22	91.7
**GP Site 4**	28	1	6	21	75	**GP Site 5**	23	2	3	18	75.0
**NW Site 1**	22	0	1	21	95.5	**GP Site 3**	26	2	2	22	84.6
**NW Site 2**	25	0	7	18	72	**GP Site 6**	24	0	1	23	95.8
**NW Site 5**	24	0	5	19	79.2	**LP Site 1**	24	0	3	21	87.5
**NW Site 3**	24	1	4	19	79.2	**LP Site 4**	24	0	0	24	100
**NW Site 6**	24	0	5	19	79.2	**LP Site 2**	24	0	2	22	91.7
**KZN Site 1**	24	0	2	22	91.7	**LP Site 5**	24	1	1	22	91.7
**KZN Site 2**	26	2	4	20	76.9	**LP Site 3**	24	1	1	22	91.7
**KZN Site 5**	27	2	3	22	81.5	**LP Site 6**	24	0	1	23	95.8
						**NW Site 4**	19	1	9	9	47.4
						**KZN Site 4**	27	0	2	25	92.6
						**KZN Site 3**	32	0	6	26	81.3
						**KZN Site 6**	26	0	4	22	84.6
**Total**	232	6	37	189	81.5	**Total**	345	7	37	301	87.2
RD**	−5.8% (−11.7% to 0.2%)										
RD in the preperiod**	0.3% (−0.5% to 1.1%)										
DiD**	−6.0% (−10.6% to −1.5%)										
DiD (cluster adjusted)***	−6.0% (−12.7% to 1.0%)										
DiD (covariate adjusted and cluster adjusted)***	−5.9% (−12.5% to 0.8%)										

*DiD analysis compares the enrolled cohort to all those who would have been eligible for DMD in the period prior to the rollout of the interventions (Jan 1, 2015 through Dec 31, 2015) (preperiod). Note that one individual was not able to be linked to TIER.Net and was not found during file review, so they do not have a retention outcome.

**Note that this is a crude analysis, with no adjustment for clustering or covariates as is done below for the final model.

***Analyses are adjusted for clustering by site using a GEE with site-level clustering and an unstructured correlation matrix; note that sample size is smaller for the DiD covariate adjusted because those with missing data will drop out of the analysis.

**Abbreviations:** DiD, difference in differences; DMD, Decentralized Medication Delivery; GEE, generalized estimating equation; GP, Gauteng Province; KZN, KwaZulu Natal; LP, Limpopo Province; LTF, Lost to follow-up; NW, North West; RD, risk difference.

## Discussion

In one of the first randomized trials of decentralized service delivery for HIV care, we found that AC patients had higher 1-year retention (89.5% versus 81.6%) and comparable sustained 1-year viral suppression (80.0% versus 79.6%) compared to those receiving standard of care. The retention association we observed was stronger for men than women (men RD: 13.1%, 95% CI: 0.3% to 23.5%; women RD: 6.0%, 95% CI: −0.9% to 12.9%). For DMD, we found that those in DMD had apparently lower retention (81.5% versus 87.2%) and comparable viral suppression compared to patients receiving standard of care (77.2% versus 74.3%), though our findings were imprecise. We also found apparently increased suppression among men (RD: 11.1%; 95% CI: −3.4% to 25.5%). Thus, our findings demonstrate that such approaches can be implemented with potential for benefit and, at a minimum, no harm among patients who already have high rates of viral suppression and retention.

South Africa’s National AGLs were developed to improve treatment outcomes and reduce the burden on clinically stable patients while allowing for provision of more intense care to individuals with advanced HIV disease. For repeat prescription strategies like DMD and for adherence approaches like ACs that also include repeat scripting, even if outcomes remain comparable to the control group, this could be seen as a benefit because these interventions are designed to make care easier for those clinically stable patients and help clinicians and staff to provide increased support to clients with advanced HIV disease and for those on a failing ART regimen, as well as freeing up capacity [31]. In fact, we might expect that the benefits of these approaches would at best be small because these interventions are designed for patients who already have high suppression and retention. We previously showed ACs were preferred by patients [32]. Here, we found ACs supported sustained viral suppression with comparable sustained viral suppression (RD: 3.8%; 95% CI: −6.9% to 14.4%) and an increase in 12-month retention (RD: 8.3%; 95% CI: 1.1% to 15.6%). Given that comparable outcomes would be acceptable for ACs, this finding, combined with our early outcomes, suggests ACs are effective. Our findings are in line with previous work that has also shown benefits to ACs [33–40]. Some have noted that ACs are inexpensive to implement and save patients time [41], and others have shown they are popular with patients [42,43]. Studies in Cape Town have shown retention to be over 90% after 12 months in clubs [21,44–47], much like in our sample [20]. The same has been shown in Kenya [19]. While these studies lacked comparison groups, in Khayelitsha, South Africa, ACs were associated with about a 12% increase in 12-month retention [22], slightly larger, though in line with, what we found. Unlike previous work, ours is one of the first to use at least some randomization for ACs (we were not able to do so for DMD), and this, combined with our DiD approach, allowed us to draw stronger conclusions. Still, caution is needed in taking the results to scale, as experience from the Western Cape has shown. There, qualitative research has shown that moving beyond the pilot phase came with complex challenges, and sites were less likely to see the benefits when the intervention was used on a large scale [48].

We did not look at short-term medication pick-up outcomes for DMD because data on pick-ups were kept by private providers and were not always fed back into patient records. Here, we looked at long-term outcomes, one of which (sustained viral suppression) did not rely on data from outside service providers. DMD was more difficult to evaluate because not all sites that were supposed to implement DMD did so, and some that were not supposed to did. Still, we found no differences in sustained viral suppression between study arms (RD: −1.0%; 95% CI: −12.2% to 10.1%). This could indicate a benefit of the intervention because it frees up clinic space. We did observe an increase in attrition associated with the intervention (RD: −5.9%; 95% CI: −12.5% to 0.8%), but we suspect this is unlikely to reflect real differences given that retention was likely influenced by visit information not being returned to clinics files. Thus, the fact that we saw only small differences suggests retention is likely unchanged. Overall, DMD appears to be a useful intervention. Unlike ACs, which have been implemented in numerous settings, there is little evidence on DMD with which to compare our data.

The evaluation was designed to evaluate the AGLs as implemented compared to standard of care. The use of a cluster-randomized evaluation helped increase validity, and use of routine data collection helped prevent the study influencing retention and suppression-based outcomes. At the same time, these choices also come with important limitations. First, it is critical when interpreting these results to understand that the control sites were not pure control groups because forms of the interventions were being implemented at many control sites. This would likely move estimates towards the null. Second, unlike individually randomized trials, our cluster-randomized design reduced power and increased the chances of confounding. Our DiD approach should help mitigate this, but some residual confounding may be left. Third, our use of routine data led to some missing data that we could not prevent. Fourth, not all those eligible for the intervention necessarily received it, and if those at intervention sites were a select group of patients, this could create bias. In addition, because subjects were not offered the interventions at control sites, we could not determine who would have received them if they had been offered. This could lead to some lack of comparability in our study populations at baseline. Fifth, while we did initially randomize clusters, this is not a fully randomized trial, both because for the DMD intervention, randomization was not maintained, but also because in the intervention sites, we only enrolled subjects who received the intervention, whereas in the control sites, we could only enroll a sample of those eligible for the interventions, even if they never would have received them if offered. The impact of this is not clear. Sixth, we note that those in the intervention arm had been on treatment for substantially longer than the control arm. We suspect this may be because providers prioritized targeting the interventions towards those who were on treatment far longer than the minimum required and had the longest track record of demonstrating stability on treatment. This could have the effect of making the interventions seem better than they are. Seventh, we conducted the study during the rollout, before sites had experience with the interventions, and as such, results under full implementation may differ. Eighth, DMD is largely run by parties outside the clinic so that data collection on the interventions was, during the study period, often controlled by third parties and did not always make it back into the data collection systems at the sites. This could have affected our long-term retention outcome. Ninth, we note that we did not a priori decide to stratify our analyses by sex, and therefore, sex-stratified results should be considered hypothesis generating and require confirmation. Finally, because these interventions were implemented at test sites, we do not know for sure that the results will generalize to all clinics in South Africa.

In conclusion, we found that ACs and DMD, in agreement with other studies, are feasible, acceptable, and for ACs, had positive outcomes. We saw an overall retention benefit to ACs and comparable outcomes with DMD against standard of care, which should still prove to be a benefit to clinics as they are designed to decongest the clinic overall. ACs as a medication refill model worked especially for male patients, given the benefits seen in retention and sustained viral suppression. As the AGL intervention package is taken to scale, it will be important to track patient retention and sustained viral suppression and to quantify the cumulative cost benefit of the package of interventions across the HIV care cascade.

## Supporting information

S1 CONSORT Checklist CONSORT 2010 checklist of information to include when reporting a cluster-randomized trial. (DOCX)

S1 Table Population data (facility headcount and total active patients) at each facility and total numbers eligible by intervention (I) and control (C) for each intervention. (DOCX)

S2 Table Viral suppression at 12 months (defined as within 2–18 months) for all those who would have been eligible for ACs in the period prior to the rollout of the interventions (Jan 1, 2015 through Dec 31, 2015) (preperiod). AC, Adherence Club.
(DOCX)

S3 Table Regression coefficients for final model for DiD analysis of AC viral suppression at 12 months (defined as within 2–18 months) adjusted for site-level clustering. AC, Adherence club; DiD, difference in differences.
(DOCX)

S4 Table Retention (alive and in care) at 12 months for all those who would have been eligible for ACs in the period prior to the rollout of the interventions (Jan 1, 2015 through Dec 31, 2015) (preperiod). AC, Adherence Club.
(DOCX)

S5 Table Regression coefficients for final model for DiD analysis of AC retention within 12 months adjusted for site-level clustering. AC, Adherence Club; DiD, difference in differences.
(DOCX)

S6 Table Effects for retention (alive and in care) at 12 months for those eligible for ACs during the intervention period (enrolled subjects only) by sex and age. AC, Adherence Club.
(DOCX)

S7 Table Viral suppression at 12 months (defined as within 2–18 months) for all those who would have been eligible for DMD in the period prior to the rollout of the interventions (Jan 1, 2015 through Dec 31, 2015) (preperiod). DMD, Decentralized Medication Delivery.
(DOCX)

S8 Table Regression coefficients for final model for DiD analysis of DMD viral suppression at 12 months (defined as within 2–18 months) adjusted for site-level clustering. DiD, difference in differences; DMD, Decentralized Medication Delivery.
(DOCX)

S9 Table Associations for viral suppression at 12 months (defined as within 2–18 months) for those eligible for DMD during the intervention period (enrolled subjects only) by sex. DMD, Decentralized Medication Delivery.
(DOCX)

S10 Table Retention (alive and in care) at 12 months for all those who would have been eligible for DMD in the period prior to the rollout of the interventions (Jan 1, 2015 through Dec 31, 2015) (preperiod). DMD, Decentralized Medication Delivery.
(DOCX)

S11 Table Regression coefficients for final model for DiD analysis of DMD retention at 12 months adjusted for site-level clustering. DiD, difference in differences; DMD, Decentralized Medication Delivery.
(DOCX)

S12 Table Associations for retention (alive and in care) at 12 months for those eligible for DMD during the intervention period (enrolled subjects only) by sex. DMD, Decentralized Medication Delivery.
(DOCX)

S1 Text Research Protocol: Evaluation of the NDOH's National AGLs for Chronic Diseases in South Africa Using Routinely Collected Data. AGL, Adherence Guideline; NDOH, National Department of Health.
(DOCX)

## Abbreviations

AC: Adherence Club
AGL: Adherence Guideline
aRD: adjusted risk difference
ART: antiretroviral therapy
CCMDD: Centralized Chronic Medicine Dispensing and Distribution
DiD: difference in differences
DMD: Decentralized Medication Delivery
GEE: generalized estimating equation
HREC: Human Research Ethics Committee
IRB: Institutional Review Board
NDOH: National Department of Health
NGO: nongovernmental organization
NHLS: National Health Laboratory Service
PHC: primary healthcare clinic
PuP: pick-up point
TB: tuberculosis
