# Supplementary material for: Adherence clubs and decentralized medication delivery to support patient retention and sustained viral suppression in care: Results from a cluster-randomized evaluation of differentiated ART delivery models in South Africa
Source: PLoS Med. 2019 Jul 23;16(7):e1002874. doi: 10.1371/journal.pmed.1002874 (PMC6650049; doi:10.1371/journal.pmed.1002874)
Supplement: S1 Table — (DOCX) [file pmed.1002874.s002.docx]

**S1 Table - Population data (facility headcount and total active patients) at each facility and total numbers eligible by intervention (I) and control (C) for each intervention**

| **Facility** | **2015 catchment population** | **Total PHC headcount, monthly 2015 average** | **Total active on ART on**  **30 June 2016**  **N** | **FTIC eligible June 1-30**  **N** | **AC/DMD eligible**  **30 June 2016**  **N (%)*** | | **EAC eligible June 1-30**  **N (%)** | | **TRIC eligible**  **30 June2016**  **N (%)** | |
| --- | --- | --- | --- | --- | --- | --- | --- | --- | --- | --- |
| **Gauteng** |  |  |  |  |  |  |  |  |  |  |
| GP 1 | 12681 | 2701 | 1563 | 43 | 486 | (31%) | 53 | (3%) | 86 | (6%) |
| GP 4 | 18686 | 3980 | 1741 | 18 | 366 | (21%) | 8 | (0%) | 374 | (21%) |
| GP 2 | 70047 | 14920 | 3434 | 17 | 1422 | (41%) | 97 | (3%) | 259 | (8%) |
| GP 5 | 76257 | 16242 | 3502 | 63 | 999 | (29%) | 146 | (4%) | 190 | (5%) |
| GP 3 | 26255 | 5592 | 2587 | 22 | 808 | (31%) | 66 | (3%) | 185 | (7%) |
| GP 6 | 21856 | 4655 | 1614 | 35 | 528 | (33%) | 55 | (3%) | 121 | (7%) |
| **Gauteng Total** | **225782** | **48090** | **14441** | **198** | **4609** | **(32%)** | **425** | **(3%)** | **1215** | **(8%)** |
|  |  |  |  |  |  |  |  |  |  |  |
| **Limpopo** |  |  |  |  |  |  |  |  |  |  |
| LP 1 | 22533 | 5 212 | 2061 | 15 | 982 | (48%) | 31 | (2%) | 158 | (8%) |
| LP 4 | 17263 | 4 511 | 1708 | 23 | 546 | (32%) | 17 | (1%) | 204 | (12%) |
| LP 2 | 25982 | 7 149 | 2184 | 30 | 621 | (28%) | 60 | (3%) | 363 | (17%) |
| LP 5 | 23644 | 5 753 | 1597 | 10 | 598 | (37%) | 16 | (1%) | 253 | (16%) |
| LP 3 | 17258 | 4265 | 1851 | 19 | 700 | (38%) | 2 | (0%) | 334 | (18%) |
| LP 6 | 22629 | 4 881 | 1132 | 10 | 233 | (21%) | 6 | (1%) | 97 | (9%) |
| **Limpopo Total** | **129309** | **31771** | **10533** | **107** | **3680** | **(35%)** | **132** | **(1%)** | **1409** | **(13%)** |
|  |  |  |  |  |  |  |  |  |  |  |
| **Northwest** |  |  |  |  |  |  |  |  |  |  |
| NW 1 | 69554 | 9825 | 3855 | 43 | 1729 | (45%) | 31 | (1%) | 487 | (13%) |
| NW 4 | 21236 | 3200 | 1848 | 49 | 875 | (47%) | 28 | (2%) | 272 | (15%) |
| NW 2 | 32352 | 4301 | 1714 | 29 | 870 | (51%) | 34 | (2%) | 130 | (8%) |
| NW 5 | 21539 | 3259 | 1431 | 24 | 751 | (52%) | 12 | (1%) | 136 | (10%) |
| NW 3 | 79290 | 15130 | 5202 | 86 | 1330 | (26%) | 50 | (1%) | 667 | (13%) |
| NW 6 | 62149 | 10810 | 3113 | 37 | 1252 | (40%) | 36 | (1%) | 520 | (17%) |
| **Northwest Total** | **286120** | **46525** | **17163** | **268** | **6807** | **(40%)** | **191** | **(1%)** | **2212** | **(13%)** |
|  |  |  |  |  |  |  |  |  |  |  |
| **KwaZulu Natal** |  |  |  |  |  |  |  |  |  |  |
| KZN 1 | 24456 | 6058 | 2528 | 28 | 1152 | (46%) | 22 | (1%) | 420 | (17%) |
| KZN 4 | 10434 | 2573 | 1089 | 11 | 609 | (56%) | 11 | (1%) | 232 | (21%) |
| KZN 2 | 42678 | 10657 | 3875 | 65 | 220 | (6%) | 15 | (0%) | 722 | (19%) |
| KZN 5 | 71060 | 18273 | 6218 | 83 | 2118 | (34%) | 6 | (0%) | 1021 | (16%) |
| KZN 3 | 21944 | 3574 | 1323 | 12 | 722 | (55%) | 10 | (1%) | 239 | (18%) |
| KZN 6 | 19103 | 3323 | 1416 | 17 | 769 | (54%) | 8 | (1%) | 99 | (7%) |
| **KwaZulu Natal Total** | **189675** | **44458** | **16449** | **216** | **5590** | **(34%)** | **72** | **(0%)** | **2733** | **(17%)** |
|  |  |  |  |  |  |  |  |  |  |  |
| **Total** | **830 886** | **170 844** | **58586** | **789** | **20686** | (35%) | **820** | (1%) | **7569** | (13%) |

*Percent of total active on ART, 30 June 2016
