# Supplementary material for: Adherence clubs and decentralized medication delivery to support patient retention and sustained viral suppression in care: Results from a cluster-randomized evaluation of differentiated ART delivery models in South Africa
Source: PLoS Med. 2019 Jul 23;16(7):e1002874. doi: 10.1371/journal.pmed.1002874 (PMC6650049; doi:10.1371/journal.pmed.1002874)
Supplement: S2 Table — AC, Adherence Club. (DOCX) [file pmed.1002874.s003.docx]

**S2 Table - Viral suppression at 12 months (defined as within 2-18 months) for all those who would have been eligible for Adherence Clubs in the period prior to the rollout of the interventions (Jan 1, 2015 through Dec 31, 2015) (pre-period)**

| **Intervention** |  |  |  |  |  | **Control** |  |  |  |  |
| --- | --- | --- | --- | --- | --- | --- | --- | --- | --- | --- |
| **Facility** | **N** | **No VL** | **Supressed** | **% Supressed** |  | **Facility** | **N** | **No VL** | **Supressed** | **% Supressed** |
| GP Site 1 | 265 | 31 | 180 | 67.9 |  | GP Site 4 | 281 | 53 | 167 | 59.4 |
| GP Site 2 | 1276 | 209 | 818 | 64.1 |  | GP Site 5 | 956 | 144 | 592 | 61.9 |
| GP Site 3 | 555 | 180 | 300 | 54.1 |  | GP Site 6 | 521 | 51 | 347 | 66.6 |
| LP Site 1 | 620 | 39 | 568 | 91.6 |  | LP Site 4 | 126 | 15 | 104 | 82.5 |
| LP Site 2 | 208 | 29 | 170 | 81.7 |  | LP Site 5 | 435 | 38 | 375 | 86.2 |
| LP Site 3 | 395 | 61 | 324 | 82.0 |  | LP Site 6 | 57 | 6 | 46 | 80.7 |
| NW Site 1 | 1395 | 330 | 1032 | 74.0 |  | NW Site 4 | 633 | 96 | 526 | 83.1 |
| NW Site 2 | 646 | 65 | 568 | 87.9 |  | NW Site 5 | 597 | 78 | 499 | 83.6 |
| NW Site 3 | 787 | 289 | 484 | 61.5 |  | NW Site 6 | 1006 | 209 | 775 | 77.0 |
| KZN Site 1 | 762 | 82 | 678 | 89.0 |  | KZN Site 4 | 398 | 33 | 357 | 89.7 |
| KZN Site 2 | 1208 | 143 | 1045 | 86.5 |  | KZN Site 5 | 1587 | 221 | 1346 | 84.8 |
| KZN Site 3 | 596 | 79 | 507 | 85.1 |  | KZN Site 6 | 661 | 33 | 621 | 93.9 |
| **Total** | 8713 | 1537 | 6674 | 76.6 |  | **Total** | 7258 | 977 | 5755 | 79.3 |
| **Risk difference** | -2.7% (-4.0% to -1.4%) | | |  |  |  |  |  |  |  |
