# Supplementary material for: Adherence clubs and decentralized medication delivery to support patient retention and sustained viral suppression in care: Results from a cluster-randomized evaluation of differentiated ART delivery models in South Africa
Source: PLoS Med. 2019 Jul 23;16(7):e1002874. doi: 10.1371/journal.pmed.1002874 (PMC6650049; doi:10.1371/journal.pmed.1002874)
Supplement: S3 Table — AC, Adherence club; DiD, difference in differences. (DOCX) [file pmed.1002874.s004.docx]

**S3 Table – Regression coefficients for final model for difference-in-differences analysis of Adherence Club viral suppression at 12 months (defined as within 2-18 months) adjusted for site level clustering***

| **Generalized Estimating Equation Parameter Estimates** | | | | | |
| --- | --- | --- | --- | --- | --- |
| **Parameter** |  | **Beta** | **Standard Error** | **95% Confidence Limits** | |
| **Intercept (% suppression in the control group in the pre-period)** |  | 0.7744 | 0.0325 | 0.7108 | 0.8381 |
| **Intervention (vs control in the pre-period)** |  | 0.0538 | 0.0459 | -0.036 | 0.1437 |
| **Post- vs pre-period (among the controls)** |  | -0.025 | 0.0473 | -0.1178 | 0.0678 |
| **intervention*period (difference-in-differences estimate)** |  | 0.0376 | 0.0541 | -0.0685 | 0.1437 |
| **Female vs. Male** |  | 0.0329 | 0.0108 | 0.0117 | 0.0541 |
| **Age 18-29.9 vs ≥ 50 years** |  | -0.0153 | 0.0174 | -0.0495 | 0.0188 |
| **Age 30-49.9 vs ≥ 50 years** |  | -0.016 | 0.0073 | -0.0303 | -0.0016 |
| **ART initiation CD4 < 200 vs ≥ 350** |  | 0.0667 | 0.0223 | 0.023 | 0.1105 |
| **ART initiation CD4 200-349 vs ≥ 350** |  | -0.0698 | 0.0414 | -0.151 | 0.0113 |

* Note that the effective sample size is decreased due to missing values for CD4 count and WHO Stage. Site level clustering adjusted for using a generalized estimating equation with an unstructured correlation matrix.
