# Supplementary material for: Adherence clubs and decentralized medication delivery to support patient retention and sustained viral suppression in care: Results from a cluster-randomized evaluation of differentiated ART delivery models in South Africa
Source: PLoS Med. 2019 Jul 23;16(7):e1002874. doi: 10.1371/journal.pmed.1002874 (PMC6650049; doi:10.1371/journal.pmed.1002874)
Supplement: S5 Table — AC, Adherence Club; DiD, difference in differences. (DOCX) [file pmed.1002874.s006.docx]

**S5 Table – Regression coefficients for final model for difference-in-differences analysis of Adherence Club retention within 12 months adjusted for site level clustering***

| **Generalized Estimating Equation Parameter Estimates** | | | | | |
| --- | --- | --- | --- | --- | --- |
| **Parameter** |  | **Beta** | **Standard Error** | **95% Confidence Limits** | |
| **Intercept (% retention in the control group in the pre-period)** |  | 0.9195 | 0.0078 | 0.9042 | 0.9349 |
| **Intervention (vs control in the pre-period)** |  | -0.057 | 0.0372 | -0.1299 | 0.0159 |
| **Post- vs pre-period (among the controls)** |  | 0.0046 | 0.0097 | -0.0145 | 0.0237 |
| **intervention*period (difference-in-differences estimate)** |  | 0.0833 | 0.0368 | 0.0112 | 0.1555 |
| **Female vs. Male** |  | 0.0159 | 0.0058 | 0.0045 | 0.0273 |
| **Age 18-29.9 vs ≥ 50 years** |  | -0.0425 | 0.0089 | -0.0599 | -0.0251 |
| **Age 30-49.9 vs ≥ 50 years** |  | -0.0132 | 0.0037 | -0.0204 | -0.0059 |
| **ART initiation CD4 < 200 vs ≥ 350** |  | 0.0173 | 0.0084 | 0.0007 | 0.0338 |
| **ART initiation CD4 200-349 vs ≥ 350** |  | -0.093 | 0.0428 | -0.1769 | -0.0091 |

* Note that the effective sample size is decreased due to missing values for CD4 count and WHO Stage. Site level clustering adjusted for using a generalized estimating equation with an unstructured correlation matrix.
