# Supplementary material for: Adherence clubs and decentralized medication delivery to support patient retention and sustained viral suppression in care: Results from a cluster-randomized evaluation of differentiated ART delivery models in South Africa
Source: PLoS Med. 2019 Jul 23;16(7):e1002874. doi: 10.1371/journal.pmed.1002874 (PMC6650049; doi:10.1371/journal.pmed.1002874)
Supplement: S6 Table — AC, Adherence Club. (DOCX) [file pmed.1002874.s007.docx]

**S6 Table - Effects for retention (alive and in care) at 12 months for those eligible for Adherence Clubs during the intervention period (enrolled subjects only) by sex and age**

| **Effects of AC in the intervention period** |  |
| --- | --- |
| **12-month follow-up retention effect among:** | **Difference** |
| **Sex** |  |
| Women | 6.0% (-0.9% to 12.9%) |
| Men | 13.1% (0.3% to 23.5%) |
| **Age** |  |
| 18-29 years | -1.2% (-13.2% to 10.8%) |
| 30-39 years | 12.3% (2.6% to 22%) |
| 40-49 years | 5.9% (-2.7% to 14.4%) |
| 50+ years | 10.8% (0.9% to 20.6%) |
