# Supplementary material for: Adherence clubs and decentralized medication delivery to support patient retention and sustained viral suppression in care: Results from a cluster-randomized evaluation of differentiated ART delivery models in South Africa
Source: PLoS Med. 2019 Jul 23;16(7):e1002874. doi: 10.1371/journal.pmed.1002874 (PMC6650049; doi:10.1371/journal.pmed.1002874)
Supplement: S7 Table — DMD, Decentralized Medication Delivery. (DOCX) [file pmed.1002874.s008.docx]

**S7 Table - Viral suppression at 12 months (defined as within 2-18 months) for all those who would have been eligible for Decentralized Medication Delivery in the period prior to the rollout of the interventions (Jan 1, 2015 through Dec 31, 2015) (pre-period)**

| **DMD Implemented** |  |  |  |  |  | **DMD Not Implemented** |  |  |  |  |
| --- | --- | --- | --- | --- | --- | --- | --- | --- | --- | --- |
| **Facility** | **N** | **No VL** | **Supressed** | **% Supressed** |  | **Facility** | **N** | **No VL** | **Supressed** | **% Supressed** |
| **GP Site 1** | 265 | 31 | 180 | 67.9 |  | **GP Site 2** | 1276 | 209 | 818 | 64.1 |
| **GP Site 4** | 281 | 53 | 167 | 59.4 |  | **GP Site 5** | 956 | 144 | 592 | 61.9 |
| **NW Site 1** | 1395 | 330 | 1032 | 74.0 |  | **GP Site 3** | 555 | 180 | 300 | 54.1 |
| **NW Site 2** | 646 | 65 | 568 | 87.9 |  | **GP Site 6** | 521 | 51 | 347 | 66.6 |
| **NW Site 5** | 597 | 78 | 499 | 83.6 |  | **LP Site 1** | 620 | 39 | 568 | 91.6 |
| **NW Site 3** | 787 | 289 | 484 | 61.5 |  | **LP Site 4** | 126 | 15 | 104 | 82.5 |
| **NW Site 6** | 1006 | 209 | 775 | 77.0 |  | **LP Site 2** | 208 | 29 | 170 | 81.7 |
| **KZN Site 1** | 762 | 82 | 678 | 89.0 |  | **LP Site 5** | 435 | 38 | 375 | 86.2 |
| **KZN Site 2** | 1208 | 143 | 1045 | 86.5 |  | **LP Site 3** | 395 | 61 | 324 | 82.0 |
| **KZN Site 5** | 1587 | 221 | 1346 | 84.8 |  | **LP Site 6** | 57 | 6 | 46 | 80.7 |
|  |  |  |  |  |  | **NW Site 4** | 633 | 96 | 526 | 83.1 |
|  |  |  |  |  |  | **KZN Site 5** | 398 | 33 | 357 | 89.7 |
|  |  |  |  |  |  | **KZN Site 3** | 596 | 79 | 507 | 85.1 |
|  |  |  |  |  |  | **KZN Site 6** | 661 | 33 | 621 | 93.9 |
| **Total** | 8534 | 1501 | 6774 | 79.4 |  | **Total** | 7437 | 1013 | 5655 | 76.0 |
| **Risk difference** | 3.3% (2.0% to 4.6%) | | |  |  |  |  |  |  |  |
