# Supplementary material for: Adherence clubs and decentralized medication delivery to support patient retention and sustained viral suppression in care: Results from a cluster-randomized evaluation of differentiated ART delivery models in South Africa
Source: PLoS Med. 2019 Jul 23;16(7):e1002874. doi: 10.1371/journal.pmed.1002874 (PMC6650049; doi:10.1371/journal.pmed.1002874)
Supplement: S8 Table — DiD, difference in differences; DMD, Decentralized Medication Delivery. (DOCX) [file pmed.1002874.s009.docx]

**S8 Table – Regression coefficients for final model for difference-in-differences analysis of Decentralized Medication Delivery viral suppression at 12 months (defined as within 2-18 months) adjusted for site level clustering***

| **Generalized Estimating Equation Parameter Estimates** | | | | | | |
| --- | --- | --- | --- | --- | --- | --- |
| **Parameter** |  | **Beta** | **Standard Error** | **95% Confidence Limits** | |  |
| **Intercept (% suppression in the control group in the pre-period)** |  | 0.7429 | 0.044 | 0.6566 | 0.8291 |  |
| **Intervention (vs control in the pre-period)** |  | 0.0425 | 0.0534 | -0.0622 | 0.1472 |  |
| **Post- vs pre-period (among the controls)** |  | 0.0385 | 0.0476 | -0.0548 | 0.1317 |  |
| **Intervention*period (difference-in-differences estimate)** |  | -0.0101 | 0.0569 | -0.1215 | 0.1013 |  |
| **Female vs. Male** |  | 0.0289 | 0.0115 | 0.0063 | 0.0514 |  |
| **Age 18-29.9 vs ≥ 50 years** |  | -0.0177 | 0.0185 | -0.0539 | 0.0185 |  |
| **Age 30-49.9 vs ≥ 50 years** |  | -0.0166 | 0.0065 | -0.0293 | -0.0039 |  |
| **ART initiation CD4 < 200 vs ≥ 350** |  | 0.0756 | 0.0234 | 0.0298 | 0.1215 |  |
| **ART initiation CD4 200-349 vs ≥ 350** |  | -0.0657 | 0.0281 | -0.1207 | -0.0106 |  |

* Note that the effective sample size is decreased due to missing values for CD4 count and WHO Stage. Site level clustering adjusted for using a generalized estimating equation with an unstructured correlation matrix.
