# Supplementary material for: Adherence clubs and decentralized medication delivery to support patient retention and sustained viral suppression in care: Results from a cluster-randomized evaluation of differentiated ART delivery models in South Africa
Source: PLoS Med. 2019 Jul 23;16(7):e1002874. doi: 10.1371/journal.pmed.1002874 (PMC6650049; doi:10.1371/journal.pmed.1002874)
Supplement: S10 Table — DMD, Decentralized Medication Delivery. (DOCX) [file pmed.1002874.s011.docx]

**S10 Table - Retention (alive and in care) at 12 months for all those who would have been eligible for Decentralized Medication Delivery in the period prior to the rollout of the interventions (Jan 1, 2015 through Dec 31, 2015) (pre-period)**

| **DMD Implemented** |  |  |  |  |  | **DMD Not Implemented** |  |  |  |  |  |
| --- | --- | --- | --- | --- | --- | --- | --- | --- | --- | --- | --- |
| **Facility** | **N** | **Transfer** | **Died/LTF** | **Alive** | **% retained** | **Facility** | **N** | **Transfer** | **Died/LTF** | **Alive** | **% retained** |
| **GP Site 1** | 265 | 11 | 11 | 243 | 91.7 | **GP Site 2** | 1276 | 89 | 55 | 1132 | 88.7 |
| **Gp Site 4** | 281 | 9 | 22 | 250 | 89.0 | **GP Site 5** | 956 | 37 | 58 | 861 | 90.1 |
| **NW Site 1** | 1395 | 21 | 39 | 1335 | 95.7 | **GP Site 3** | 555 | 12 | 40 | 503 | 90.6 |
| **NW Site 2** | 646 | 26 | 22 | 598 | 92.6 | **GP Site 6** | 521 | 18 | 24 | 479 | 91.9 |
| **NW Site 5** | 597 | 30 | 27 | 540 | 90.5 | **LP Site 1** | 620 | 13 | 18 | 589 | 95.0 |
| **NW Site 3** | 787 | 8 | 46 | 733 | 93.1 | **LP Site 4** | 126 | 7 | 2 | 117 | 92.9 |
| **NW Site 6** | 1006 | 29 | 41 | 936 | 93.0 | **LP Site 2** | 208 | 1 | 9 | 198 | 95.2 |
| **KZN Site 1** | 762 | 19 | 47 | 696 | 91.3 | **LP Site 5** | 435 | 2 | 7 | 426 | 97.9 |
| **KZN Site 2** | 1208 | 28 | 73 | 1107 | 91.6 | **LP Site 3** | 395 | 18 | 12 | 365 | 92.4 |
| **KZN Site 5** | 1587 | 66 | 69 | 1452 | 91.5 | **LP Site 6** | 57 | 2 | 0 | 55 | 96.5 |
|  |  |  |  |  |  | **NW Site 4** | 633 | 59 | 11 | 563 | 88.9 |
|  |  |  |  |  |  | **KZN Site 5** | 398 | 6 | 14 | 378 | 95.0 |
|  |  |  |  |  |  | **KZN Site 3** | 596 | 20 | 16 | 560 | 94.0 |
|  |  |  |  |  |  | **KZN Site 6** | 661 | 20 | 13 | 628 | 95.0 |
| **Total** | 8534 | 247 | 397 | 7890 | 92.5 | **Total** | 7437 | 304 | 279 | 6854 | 92.2 |
| **Risk difference** | 0.3% (-0.5% to 1.1%) | | |  |  |  |  |  |  |  |  |
