# Supplementary material for: Adherence clubs and decentralized medication delivery to support patient retention and sustained viral suppression in care: Results from a cluster-randomized evaluation of differentiated ART delivery models in South Africa
Source: PLoS Med. 2019 Jul 23;16(7):e1002874. doi: 10.1371/journal.pmed.1002874 (PMC6650049; doi:10.1371/journal.pmed.1002874)
Supplement: S11 Table — DiD, difference in differences; DMD, Decentralized Medication Delivery. (DOCX) [file pmed.1002874.s012.docx]

**S11 Table – Regression coefficients for final model for difference-in-differences analysis of Decentralized Medication Delivery retention at 12 months adjusted for site level clustering***

| **Generalized Estimating Equation Parameter Estimates** | | | | | | |
| --- | --- | --- | --- | --- | --- | --- |
| **Parameter** |  | **Beta** | **Standard Error** | **95% Confidence Limits** | |  |
| **Intercept (% retention in the control group in the pre-period)** |  | 0.9215 | 0.0113 | 0.8993 | 0.9436 |  |
| **Intervention (vs control in the pre-period)** |  | 0.0189 | 0.0295 | -0.0389 | 0.0768 |  |
| **Post- vs pre-period (among the controls)** |  | 0.0048 | 0.0102 | -0.0151 | 0.0247 |  |
| **Intervention*period (difference-in-differences estimate)** |  | -0.0589 | 0.0339 | -0.1253 | 0.0075 |  |
| **Female vs. Male** |  | 0.0127 | 0.0058 | 0.0014 | 0.0241 |  |
| **Age 18-29.9 vs ≥ 50 years** |  | -0.042 | 0.0085 | -0.0588 | -0.0253 |  |
| **Age 30-49.9 vs ≥ 50 years** |  | -0.0129 | 0.0037 | -0.0203 | -0.0056 |  |
| **ART initiation CD4 < 200 vs ≥ 350** |  | 0.0179 | 0.0086 | 0.0011 | 0.0347 |  |
| **ART initiation CD4 200-349 vs ≥ 350** |  | -0.046 | 0.0322 | -0.109 | 0.0171 |  |

* Note that the effective sample size is decreased due to missing values for CD4 count and WHO Stage. Site level clustering adjusted for using a generalized estimating equation with an unstructured correlation matrix.
