# Supplementary material for: Adherence clubs and decentralized medication delivery to support patient retention and sustained viral suppression in care: Results from a cluster-randomized evaluation of differentiated ART delivery models in South Africa
Source: PLoS Med. 2019 Jul 23;16(7):e1002874. doi: 10.1371/journal.pmed.1002874 (PMC6650049; doi:10.1371/journal.pmed.1002874)
Supplement: S12 Table — DMD, Decentralized Medication Delivery. (DOCX) [file pmed.1002874.s013.docx]

**S12 Table. Associations for retention (alive and in care) at 12 months for those eligible for Decentralized Medication Delivery during the intervention period (enrolled subjects only) by sex***

| **Effects of DMD in the intervention period** |  |
| --- | --- |
| **12-month follow-up retention effect among:** | **Difference** |
| **Sex** |  |
| Women | -3.6% (-10.3% to 3.1%) |
| Men | -12.0% (-24.2% to 0.1%) |

* Note these are crude comparison in the post period only, not difference-in-differences adjusted.
