# Supplementary material for: Adherence clubs and decentralized medication delivery to support patient retention and sustained viral suppression in care: Results from a cluster-randomized evaluation of differentiated ART delivery models in South Africa
Source: PLoS Med. 2019 Jul 23;16(7):e1002874. doi: 10.1371/journal.pmed.1002874 (PMC6650049; doi:10.1371/journal.pmed.1002874)
Supplement: S1 Text — AGL, Adherence Guideline; NDOH, National Department of Health. (DOCX) [file pmed.1002874.s014.docx]

**S1 Text - Research Protocol**

**Evaluation of the National Department of Health's**

**National Adherence Guidelines for Chronic Diseases in South Africa**

**Using Routinely Collected Data**

**October 19, 2016**

Version 3.0

Wits Protocol No.: M150537

**CONTENTS**

1. SUMMARY 5

2. INVESTIGATORS 5

3. BACKGROUND, RATIONALE, AND OBJECTIVES 8

**a. Background** 8

**b. Rationale** 8

**c. Objectives** 9

4. EVALUATION OF INTERVENTION EFFECTIVENESS (OBJECTIVES 1-5) 11

**a. Fast Track ART Initiation Counseling** 12

**b. Adherence Clubs** 13

**c. Decentralized Medication Delivery** 13

**d. Enhanced Adherence Counseling** 14

**e. Early Tracing of Patients Lost to Follow Up** 14

5. ADHERENCE STRATEGY IMPACT (OBJECTIVE 6) 15

6. COST (OBJECTIVE 7) 15

7. TUBERCULOSIS, HYPERTENSION, AND DIABETES (OBJECTIVE 8) 15

8. STUDY SITES AND POPULATION 16

**a. Selection and Randomization of Study Sites** 16

**b. Inclusion and Exclusion Criteria** 17

9. DATA SOURCES AND MANAGEMENT 18

**a. Data Sources** 18

**b. Data Fields to be collected** 19

**c. Duration of Follow Up** 21

**d. Data Entry and Storage** 22

10. DATA ANALYSIS 23

**a. Outcomes** 23

**b. Sample Size** 25

**c. Data Analysis Objectives 1-5** 27

**d. Data Analysis Objective 6** 27

**e. Data Analysis Objective 7** 27

**f. Data Analysis Objective 8** 28

**g. Dissemination of Findings** 28

11. ETHICAL CONSIDERATIONS 28

**a. Potential Risks and Protections** 28

**b. Direct Benefits** 29

**c. Indirect (Societal) Benefits** 29

**d. Informed Consent** 30

**e. Subject Confidentiality** 30

**f. Costs and Payments** 30

12. REFERENCES 30

**APPENDICES**

I. National Adherence Guidelines for Chronic Diseases (HIV, TB and NCDs) (version of 7 April 2015)

II. Adherence Plan

III. Chronic Patient Record

# SUMMARY

The South Africa National Department of Health (NDOH) intends to launch its newly developed National Adherence Guidelines for Chronic Diseases (HIV, TB and NCDs) throughout South Africa in the coming year. Early implementation of the “minimum package” of interventions described in the Adherence Guidelines for HIV patients will take place at 12 primary health clinics and community health centres in four provinces starting in July 2015. To maximize the learning potential of this early implementation stage, NDOH will match the intervention clinics with 12 comparison clinics and randomly allocate intervention or comparison status within the pairs of clinics. This will allow the outcomes of the interventions to be evaluated using a cluster-randomized design and generate data on the costs of implementation and the potential need for adherence support for the other diseases addressed in the guidelines (tuberculosis, hypertension, and diabetes). This protocol is for the evaluation, which will generate information on the effectiveness of minimum package interventions and help improve the design, implementation, and budgeting of the guidelines.

We will assess the effectiveness of five interventions in the minimum package: 1) Fast track initiation counseling for patients eligible for antiretroviral therapy; 2) Enhanced adherence counseling for unstable patients on HIV treatment; 3) adherence clubs for stable patients on HIV treatment; 4) decentralized medication delivery for stable patients on HIV treatment; and 5) early tracing of all patients who miss an appointment by two weeks. We will also estimate for each study site an overall “adherence guideline impact” to provide an indication of the effectiveness of the package as a whole.

In addition, we will estimate the cost of each of the interventions listed above compared to standard of care. Finally, we will describe the cascade of care for tuberculosis, hypertension, and diabetes, three other chronic diseases for which little information currently exists. To evaluate these interventions we will work with the National Department of Health (which will implement the interventions) to randomize 24 clinics in 4 provinces 1:1 to receive the interventions or continue standard of care. All evaluations will use data routinely collected by the clinics, with no study interaction with subjects. A total of 8,256 patients will be enrolled and followed for up to 20 months to estimate short- and long-term outcomes.

# INVESTIGATORS

This evaluation will be conducted by investigators from Boston University (School of Public Health) and Wits University (Health Economics and Epidemiology Research Office, Wits Health Consortium), in close collaboration with the Department of Health of South Africa and the World Bank. Individual investigators are:

Boston University

*Principal investigator*

Matthew Fox

Associate Professor

Boston University School of Public Health

United States

*Co-investigator*

Sydney Rosen

Research Professor

Boston University School of Public Health

United States

*Co-investigator*

Nancy Scott

Assistant Professor

Boston University School of Public Health

South Africa

*Co-investigator*

Bruce Larson

Research Professor

Boston University School of Public Health

South Africa

HE^2^RO (Wits Health Consortium, Wits University)

*Local Lead Investigator*

Sophie Pascoe

Senior Researcher

Health Economics and Epidemiology Research Office

South Africa

Additional co-investigators from HE^2^RO may be added prior to starting the evaluation.

Several other partners will provide technical assistance in the design and implementation of the evaluation but will not have access to data or be engaged in human subjects research.

National Department of Health (not engaged in research)

Mokgadi Phokojoe

*Co-Principal investigator*

Director, HIV and AIDS Care and Support Programme

National Department of Health

South Africa

World Bank

Nicole Fraser-Hurt

*Co-Principal investigator*

M&E Specialist and South Africa Study Coordinator

The World Bank

United States

Zara Shubber

*Co-investigator*

Public Health Specialist

The World Bank

United States

Marelize Gorgens

*Co-investigator*

HIV Program Manager

The World Bank

United States

# BACKGROUND, RATIONALE, AND OBJECTIVES

## **a. Background**

For antiretroviral therapy (ART) for HIV and treatment for other chronic diseases to be effective, patients must remain in care for long periods of time, initiate treatment as early as allowed under prevailing guidelines, consistently achieve high levels of adherence to their treatment regimen and, as a result, exhibit low and stable monitoring test results and/or treatment completion. In the case of HIV, treatment is lifelong and requires consistent, nearly complete adherence to sustain an undetectable viral load. Consistent adherence is also necessary to achieve success with treatment for tuberculosis, diabetes, hypertension, and other non-communicable diseases (NCDs). Numerous studies and reviews[1–4], as well as the South African National Department of Health’s (NDOH) own data[5], have indicated that retention in care and adherence to ART in South Africa are sub-optimal and pose a serious threat to the long-term success of the national HIV response. Although there is less evidence on hand, these same problems almost certainly also pertain to tuberculosis (TB), for which treatment completion and cure rates do not approach global targets[6], and to NCDs, for which almost no treatment adherence data are available.

To address this challenge, in 2014 the NDOH developed the “National Adherence Guidelines for Chronic Diseases (HIV, TB and NCDs)”. The guidelines address the provision of a minimum package of interventions to increase linkage to care, retention in care, and adherence to treatment. The minimum package interventions are listed in Table 1.

**Table 1. National Adherence Guidelines minimum package of interventions**

| **Approach** | **Intervention** |
| --- | --- |
| Education and counselling | 1. Fast track initiation counseling* 2. Enhanced adherence counseling for unstable patients* 3. Child disclosure counseling for children living with HIV |
| Repeat prescription collection strategies | 1. Adherence clubs* 2. Spaced and fast lane appointment systems 3. Decentralised medication delivery* |
| Patient tracing | 1. Early tracing of all missed appointments* |
| Integrated HIV, TB, NCD care | 1. Integrated consultation and counselling |

*Indicates interventions included in this evaluation

## **b. Rationale**

Although there is some published or unpublished evidence of the effectiveness of each of these interventions for HIV care[7–9], most have not been implemented jointly or at scale, nor have they been evaluated as delivered routinely by public sector facilities, without external technical assistance or resource support. South Africa also lacks evidence on HIV/NCD co-morbidities and on the prevalence and current treatment outcomes of patients presenting with NCDs. Better information is needed to guide the NDOH’s rollout of the minimum package at national scale and about the number of patients requiring intervention and the cost of delivering the interventions to them.

Prior to national scale-up of the Adherence Guidelines, NDOH intends to select 12 clinics (primary health care clinics and community health centres) for early implementation of the minimum package for HIV patients. This will generate information to refine the guidelines, gain experience in implementation, and project budgetary needs. To maximize the learning potential of these early implementation sites, they will be matched with 12 other clinics and then one clinic in each pair will be randomly assigned to receive the interventions, with the other clinic in the pair serving as a comparison site. This will allow for a cluster-randomized evaluation of the impact of the interventions. The early implementation sites will also generate data on the costs of the interventions and on how to tailor and target the interventions to tuberculosis, hypertension, and diabetes. At the request of the NDOH, we previously developed a set of cost estimates for national implementation of the Adherence Guidelines using published sources of data[16]; this evaluation will also allow refinement of the cost estimates, which are needed to assure sufficient resources for large-scale delivery.

Each of the interventions listed in Table 1 addresses a unique population of patients within a clinic, with the exception of interventions 4-6, which are substitutes for one another. For example, fast track initiation counseling is limited to patients eligible to start treatment; enhanced adherence counseling is limited to patients failing treatment; early tracing of missed appointments is limited to patients who are late for scheduled visits; and so on. While individual patients may become eligible for multiple interventions over the course of their care, the interventions will not serve overlapping cohorts of patients simultaneously. This will allow the impact of each intervention to be evaluated separately, as well as within the overall package.

## **c. Objectives**

The overall aims of this study are to assess the impact of a subset of the National Adherence Guidelines’ minimum package of interventions on HIV patients’ treatment outcomes at public sector clinics; estimate the costs of the interventions; and describe the cascade of care for TB, hypertension, and diabetes at these same clinics. All implementation of the interventions and generation of data will be done by NDOH; this protocol, which has been developed in partnership with the National Department of Health, is solely for the analysis of routinely collected data.

The specific objectives of the evaluation are to:

*Objective 1: Among HIV-infected patients newly eligible for antiretroviral therapy,* ***evaluate the impact of Fast Track Treatment Initiation Counselling on initiation and viral suppression****.*

Research questions:

- What is the effect of Fast Track Initiation Counselling on rates of initiating ART within 30 days of determining ART eligibility?
- What is the effect of Fast Track Initiation Counselling on rates of being alive, in care, and virally suppressed within 9 months of determining ART eligibility?

*Objective 2: Among HIV-infected patients who are stable on antiretroviral therapy,* ***evaluate the impact of Adherence Clubs on ART adherence and viral suppression****.*

Research questions:

- What is the effect of Adherence Clubs on medication possession over the first 3 months of intervention eligibility compared to standard of care?
- What is the effect of Adherence Clubs on rates of viral suppression within 12 months of intervention eligibility compared to standard of care?

*Objective 3: Among HIV-infected patients who are stable on antiretroviral therapy,* ***evaluate the impact of Decentralized Medication Delivery on ART adherence and viral suppression****.*

Research questions:

- What is the effect of Decentralized Medication Delivery on medication possession in the first 3 months after intervention eligibility compared to standard of care?
- What is the effect of Decentralized Medication Delivery on rates of viral suppression within 12 months of intervention eligibility compared to standard of care?

*Objective 4: Among HIV-infected patients who have poor adherence to antiretroviral therapy,* ***evaluate the impact of Enhanced Adherence Counselling on treatment adherence and viral suppression****.*

Research question:

- What is the effect of Enhanced Adherence Counselling on rates of being alive, in care, and virally suppressed within 3 and 12 months of intervention eligibility?

*Objective 5: Among HIV-infected patients in antiretroviral therapy programs who miss a scheduled appointment by 5 days,* ***evaluate the impact of Early Patient Tracing on retention in care****.*

Research questions:

- What is the effect of Early Patient Tracing on rates of returning to care by 3 and 12 months of tracing?
- What is the effect of Early Patient Tracing on rates of remaining in care by 12 months of tracing?

*Objective 6: For each clinic included in the study,* ***evaluate the overall impact of the Adherence Guidelines on patient outcomes****.*

Research questions:

- What is the effect of the package of interventions on patient retention in care overall?
- What is the proportion of population in need expected to benefit from the package of interventions?

*Objective 7:* ***Estimate the incremental and total cost of each of the interventions*** *listed above compared to standard of care.*

Research questions:

- What is the average cost per patient reached with the intervention?
- What is the average cost per successful outcome achieved?

*Objective 8:* ***Describe the current status of the cascade of care and adherence to treatment for tuberculosis, hypertension, and diabete****s, for the purpose of tailoring the minimum package of interventions to these conditions in the national rollout.*

Research questions:

- What is the prevalence of previously and newly diagnosed TB, hypertension, and diabetes among the population of patients presenting for care at primary health clinics, stratified by age, sex, and HIV status where possible?
- What proportion of diagnosed cases of these conditions are initiated on guideline-compliant treatment?
- What proportion of cases have satisfactory outcomes, based on current guidelines 12 months after diagnosis?
- For each condition, what proportion of patients are lost to care or discontinue treatment during the first 12 months after diagnosis?

Note that the impacts being assessed under Objectives 1-6 are the impacts of the interventions as implemented in the early phase of the national rollout of the guidelines. As is the case with program evaluations in general, results will reflect both intervention efficacy and implementation quality, which may in turn reflect the early learning stage of national policy implementation.

Prior to starting data collection for this evaluation, we will develop descriptions of each study facility. This will include staffing, patient population, services provided, current standard of care, existing adherence-related services and programs, quality of routine data collection, support received from PEPFAR and other external partners, community organizations that refer to the clinic or support its patients, relevant published studies already completed at the sites, and other facility-level information that may help interpret the evaluation results and inform recommendations. These preparatory descriptions are not an objective of the evaluation itself and do not involve human subjects, but will provide useful insights as to the readiness of sites for implementation, challenges and gaps that need to be addressed and the implications of these on the implementation and scale-up of national guidelines.

# EVALUATION OF INTERVENTION EFFECTIVENESS (OBJECTIVES 1-5)

To achieve the first five objectives, we will test the effectiveness of five interventions included in the minimum package of adherence interventions using routinely collected data only. The interventions have been developed by the NDOH and will be implemented by the public sector clinics using their own staff and resources. Those interventions included in the evaluation were selected as having outcomes that could be assessed in a relatively short period of time, so that results can be used to refine the national guidelines and implementation. Below we detail each of the interventions and the populations we will use to evaluate the interventions.

We will use a matched cluster randomized design to evaluate the impact of each of the selected interventions. The National Department of Health, in consultation with the study team, will develop a short list of candidate primary health care clinics where early implementation of the package can occur. This short list will include proposed matched pairs of facilities which will be randomized to either implement the minimum package or not. Clinics will be matched on characteristics that are likely to affect study outcomes (patient volume, urban/rural location, current HIV viral suppression rate).

Because the interventions will be implemented clinic-wide and the evaluation will rely on routinely collected data only, we will include the entire patient population at each study site. All non-pregnant adult patients seeking HIV-related services at the study sites and eligible to receive one of the adherence interventions during the enrollment period will be included in the study through use of their clinic data. There will be no direct interaction with study subjects. Data will be collected from existing, routinely completed patient records, including clinic files, registers, cards, and databases. No additional data fields, beyond what are currently mandated under government guidelines, will be collected for the study. We assume that all study sites will follow the current guidelines for HIV care and treatment, dated December 2014[10] and the Primary Care 101 guide for TB, HIV, diabetes and hypertension care and treatment, dated 2013/14[15] as well as comply with the National Health Act of 2003.

Each of the interventions, while implemented as a package of services, will be delivered to a unique population within each clinic: patients newly eligible for ART, patients stable on ART, patients with poor adherence, and patients lost from care. (The exception to this is patients who are stable on ART and could be provided with either decentralized medication delivery or adherence clubs, but not both.) This will allow us to estimate the effect of each intervention individually, in the context of implementation of the overall minimum package. During the early stages of implementation of the Adherence Guidelines it is possible that eligible patients at intervention sites might not all be enrolled in an intervention they are eligible for. In order to ensure that this evaluation does not by chance exclude eligible patients who are exposed to the interventions, enrolment at intervention sites will include not only those eligible for the intervention but also eligible patients who receive the intervention.

The following five interventions will be evaluated under this protocol. Each is described in detail in the National Adherence Guidelines[11]. (Note: Additional details about primary and secondary outcomes, eligibility criteria, sample size, and data sources are included in later sections of this protocol.)

## **a. Fast Track ART Initiation Counseling**

Fast track initiation seeks to reduce attrition from chronic care by speeding up the process of treatment initiation for patients who are eligible for treatment and thereby increasing the proportion of treatment-eligible patients who start treatment promptly. For HIV, the goal is to reduce the total number of visits that patients need to complete in order to start treatment and allow patients to initiate treatment over the course of two clinic visits within one week of confirming ART eligibility, with additional counseling provided in the first two routine visits after treatment initiation. The intervention includes a detailed curriculum for the counseling sessions, and providers work with patients to create an individualized adherence plan and ensure post-initiation adherence support[12].

| *Primary outcome:* | Proportion of ART-eligible patients who initiate ART (obtain their first supply of ARV medications) within 30 days of determination of treatment eligibility |
| --- | --- |
| *Eligibility:* | All non-pregnant adult patients who are eligible for ART according to national guidelines (CD4 cell count ≤ 500 or Stage 3 or 4 condition) |
| *Sample size:* | 720 (360 per arm; 30 per cluster) |
| *Data sources:* | HIV counseling and testing (HCT) and CD4 count registers; adherence plans; Tier.Net (South Africa’s national HIV patient care database); counselling registers and other registers or records that document services received prior to ART initiation (including patient record) |

## **b. Adherence Clubs**

Adherence clubs comprise adherent and stable patients on ART who meet at facilities or identified locations in the community, in groups of up to 30 patients every two to three months to receive group counseling, have a clinical assessment, and receive the required supply of pre-packed medications. Adherence clubs are managed by lay staff at the health care facility with support from community health workers. The goal is to keep patients engaged in care and adherent to their medication by providing social support and facilitating medication delivery and treatment monitoring, while also reducing patient visit burden on the clinics[13]. The adherence guidelines SOP provides detailed instructions for establishing and running the clubs and for eligibility criteria and data collection.

| *Primary outcome:* | Proportion of patients eligible for participation in an adherence club who receive all medications within the first 4 months after eligibility |
| --- | --- |
| *Eligibility:* | All adult patients who meet the criteria for being stable on ART (on same ART treatment regimen for at least 12 months; most recent viral load taken in past 6 months, 2 consecutive viral loads undetectable ) OR where a clinician confirms eligibility as a stable patient |
| *Sample size:* | 576 (288 per arm; 24 per cluster) |
| *Data sources:* | Tier.Net; pharmacy records; club register; other registers or records that document services provided during the period of club observation |

## **c. Decentralized Medication Delivery**

Decentralized medication delivery through the “Centralized Chronic Medication Dispensing Distribution” (CCMDD) uses locations other than the clinic pharmacy to deliver medications to patients who are stable on treatment. Patients then only need to come to the clinic on a six monthly basis for a clinical exam. The goal is to reduce the burden on the patient in terms of the time and resources it takes them to collect their medication to improve treatment adherence and retention in care, while also decongesting the clinics.

| *Primary outcome:* | Proportion of patients eligible for decentralized medication delivery who receive all medications within the first 3 months after eligibility |
| --- | --- |
| *Eligibility:* | All adult patients who meet the criteria for being stable on ART (on same ART treatment regimen for at least 12 months; most recent viral load taken in past 6 months, 2 consecutive viral loads undetectable ) OR where a clinician confirms eligibility as a stable patient |
| *Sample size:* | 576 (288 per arm; 24 per cluster) |
| *Data sources:* | Tier.Net; pharmacy records; pickup point records or CCMDD registers or databases; other registers or records that document services provided during period of decentralized delivery |

## **d. Enhanced Adherence Counseling**

The goal of this intervention is to identify patients who have poor treatment adherence as indicated by an elevated viral load and target these patients for enhanced adherence counseling to help them improve their adherence. Although HIV-infected patients with detectable viral loads may receive additional adherence counseling under standard of care, this intervention will standardize and intensify that counseling. The intervention includes one or two structured education/counseling sessions in which effective strategies for achieving good adherence are discussed and goals set for viral re-suppression.

| *Primary outcome:* | Proportion of patients with an elevated viral load who are alive, in care and resuppress their viral load within 3 months of eligibility for enhanced adherence counseling |
| --- | --- |
| *Eligibility:* | All adult patients with a detectable viral load at a routine monitoring visit |
| *Sample size:* | 1008 (504 per arm; 42 per cluster) |
| *Data sources:* | NHLS laboratory database; Tier.Net; adherence plans; counselling registers or other registers or records that document provision of counselling |

## **e. Early Tracing of Patients Lost to Follow Up**

Early tracing of patients who miss an appointment by 5 days or more seeks to identify patients who have not returned to the clinic for scheduled appointments and attempts to return them to care. This intervention requires obtaining permission from patients to contact them and maintaining up to date contact information in patient records. The goal is to reduce clinic loss to follow up and improve patient outcomes by identifying those who have missed appointments and seeking to encourage them to return to care.

| *Primary outcome:* | Proportion of patients who miss a scheduled appointment by 5 days or more who return to care within 3 months of tracing |
| --- | --- |
| *Eligibility:* | All adult patients who fail to return for a scheduled appointment within 5 to 90 days from their appointment date |
| *Sample size:* | 576 (288 per arm; 24 per cluster) |
| *Data sources:* | Tier.Net; tracing registers or logbooks; appointment books; consent forms; other registers or records that document tracing attempts and interactions with patients |

In the early implementation phase at the study clinics, the government will also test fast track/spaced appointments for patients on antiretroviral therapy. This strategy is targeted at patients who are stable on treatment as defined above and therefore does overlap with the adherence club and decentralized medication delivery populations. If fast track/spaced appointments are implemented for the same patients enrolled in the adherence clubs or receiving decentralized medication delivery in the pilot facilities, the interventions evaluated will then be “adherence clubs plus fast tracked/spaced appointments” and “decentralized medication delivery plus fast tracked/spaced appointments,” as it will not be possible to distinguish between the impacts of the two interventions.

# ADHERENCE STRATEGY IMPACT (OBJECTIVE 6)

To capture the effect of the Adherence Guidelines as a whole, under Objective 6 an overall “adherence strategy impact” including the effects of all interventions conducted will be estimated for each study site (cluster), to provide an indication of the effectiveness of the package as a whole. This analysis will rely on the same data collected under Objectives 1-5, but will also utilize aggregate site-level reports to apply the effects of the interventions to the total population in need presenting at the evaluation clinics per year. The estimate developed for this objective will allow comparison among intervention clinics and provide NDOH with data to refine its expectations and models for long term chronic disease care.

# COST (OBJECTIVE 7)

Objective 7 calls for estimating the cost of the five interventions implemented as part of the minimum package. We will use a mix of bottom-up and top-down methods to estimate the cost per service delivered and, where data allow, the cost per outcome achieved. The same routine patient data collected for the intervention evaluation will be used to identify and quantify resource utilization per patient in care, using methods we developed for early HIV treatment studies. Unit costs will be collected from a subset of the study clinics using invoices, prices lists, and rate scales. The average cost per service delivered, per outcome achieved, per patient served, and per facility will then be estimated. If data from Objective 6 allow, we will also estimate the cost of care for tuberculosis, hypertension, and diabetes. Data and analytic methods are described in more detail below.

# TUBERCULOSIS, HYPERTENSION, AND DIABETES (OBJECTIVE 8)

Under Objective 8 we will collect baseline data to help tailor the adherence interventions to other chronic conditions in the future. This objective will focus on drug-susceptible tuberculosis, adult Type 2 diabetes mellitus, and hypertension. Routinely collected data will be used to estimate proportions of patients diagnosed with each condition, treatment uptake, and treatment retention at the same study sites in the cluster randomized trial.

The Adherence Guidelines address tuberculosis and common non-communicable diseases, like hypertension and diabetes, as well as HIV. Unlike for HIV, where much is known about the care cascade and where the “leaks” in the cascade have been identified and estimated, little is known about the care cascade for TB and even less for diabetes and hypertension. A better evidence base about current retention in care and treatment outcomes is needed to assess the magnitude of the problem and define areas most in need of targeting for adherence support before the minimum package of adherence interventions is offered to patients in these populations.

To generate this evidence, we will use routinely collected data from the same clinics at which the HIV interventions will be evaluated. From existing medical records, we will estimate the proportions of patients presenting at the clinics who are screened for TB, hypertension, and/or diabetes and those retained in care at various steps along the diagnostic and treatment cascade, stratified where data allow by HIV status, sex, age, and/or other variables. Details on specific outcomes to be estimated, sample size, and data sources are provided in later sections of this protocol.

# STUDY SITES AND POPULATION

## **a. Selection and Randomization of Study Sites**

The evaluation will be conducted at 24 primary health care clinics (PHCs) in South Africa. Six clinics will be chosen from one district each in Gauteng, KwaZulu Natal, Limpopo, and North West Provinces. These provinces have been chosen in consultation with NDOH as they are high HIV burden provinces with high burden districts and high volume clinics. Criteria for selection of clinics are:

- *High patient volume*. Although the adherence guidelines apply to multiple conditions, the impact evaluation will focus on HIV (ART initiation, retention, and adherence). To achieve adequate enrollment within the time frame of the evaluation, only sites with >1000 current ART patients will be included. (It is likely that these will be large clinics in general and thus also have relatively high volumes of TB, diabetes, and hypertension patients for Objective 8.)

- *Not already a National Health Insurance pilot site*. The NDOH is piloting a series of interventions in selected districts in preparation for the introduction of national health insurance. These districts will be excluded as they are already providing enhanced care and therefore it would be difficult to assess the impact of the minimum package interventions.
- *Generating computerized Tier.Net data*. Some clinics continue to use only paper records to record and report on ART patients. Since the evaluation will rely heavily on patient records, it will not be practical to work at sites that have not yet implemented a computer-based system. (Note that we can work at sites that are not yet networked under Tier.Net, South Africa’s national HIV patient care database, just not at those that remain paper-based.)

- *Not participating in any other adherence-related studies or pilots*. Although most clinics will already be providing some degree of adherence support to patients, under existing guidelines, a few may also be participating in research or special programmes pertaining to adherence. We would like to avoid these so that the impacts observed can be attributed to the adherence guideline interventions.

The NDOH will develop a short list of all sites in each participating province that meet these criteria. We will then assist NDOH to select three matched pairs of clinics per province. The criteria for matching are:

- *Size*. Both clinics in each pair should have either 1000-1999; 2000-4999; or ≥5000 current ART patients.
- *Setting*. Both sites in each pair should draw from the same type of community: urban; informal settlement; or rural.
- *Location*. Pairs should be located relatively nearby one another, to facilitate data collection and increase similarity of the catchment populations.

- *HIV viral suppression*. The clinics in the pair should have ART patient viral suppression rates within 10% percent of one another (if data available).

Letters indicating Provincial and District Department of Health authorization to work at each study site have been obtained and are attached. The final number of clinics selected remains at 24 clusters.

In each pair, one clinic will randomly be assigned to receive early implementation of the minimum package of interventions, while the other will continue to provide standard of care. Randomization will be done through a random number generator in Microsoft Excel with a 1:1 ratio of allocation to intervention and control clusters.

## **b. Inclusion and Exclusion Criteria**

For Objectives 1-5 and Objective 8, we will enroll a specific cohort of patients. These will be numbered in accordance with the objective number for Objectives 1-5; for Objective 8, the cohort will be called the “TBHD cohort”, in reference to tuberculosis, hypertension, and diabetes. This TBHD cohort will be made up of patients who have been screened for TBHD (50%) and patients who are newly diagnosed with TB, hypertension or diabetes (50%). For all cohorts, inclusion and exclusion criteria are as shown below. For Objectives 1-5, these criteria follow the December 2014 national guidelines for HIV care and ART[10] and November 2015 National Adherence Guidelines for Chronic Disease (HIV, TB and NCDs) [11].

*Cohorts 1-5 and TBHD cohort inclusion criteria*

- ≥ 18 years old
- Meet the inclusion criteria for one or more of Cohorts 1-8

*Cohorts 1-5 and TBHD cohort exclusion criteria*

- Not resident in the facility’s catchment area
- Recorded intention to transfer care to a different facility within 12 months
- Pregnant and eligible for PMTCT

*Objective 1/Cohort 1 inclusion criteria (Patients newly eligible for ART—fast track treatment initiation)*

- Determined to be eligible to start ART under prevailing national guidelines (CD4 count < 500, WHO Stage 3 or 4 condition, TB, cryptococcal meningitis)

*Objectives 2 and 3/Cohorts 2 and 3 inclusion criteria (Patients stable on ART—adherence clubs or decentralized medication delivery)*

- On same ART treatment regimen for at least 12 months
- Most recent viral load taken in past 6 months
- Two consecutive viral loads undetectable (<400 copies/ml^3^)

*Objective 4/Cohort 4 inclusion criteria (Patients on ART with poor adherence—enhanced adherence counseling)*

- On first line ART for at least 6 months
- Poor adherence as indicated by an elevated viral load (>400 copies/ml^3^)

*Objective 5/Cohort 5 inclusion criteria (Patients lost from ART programs—early patient tracing)*

- Initiated ART
- Fail to return for a scheduled appointment within 5 to 90 days from their appointment date

*Objective 8/TBHD cohort inclusion criteria (Tuberculosis, hypertension, and diabetes patients)*

- Screened for or diagnosed with for TB or hypertension within the last month, or diabetes within last 6 months

*Objective 8/TBHD cohort exclusion criteria (Tuberculosis, hypertension, and diabetes patients)*

- Patients with existing TB, hypertension (diagnosed more than 1 month ago) or diabetes (diagnosed more than 6 months ago)

# DATA SOURCES AND MANAGEMENT

## **a. Data Sources**

The NDOH and its partners will implement the interventions listed above as the initial component of a new national policy. This protocol is solely for an evaluation of the impact of the interventions. The study team will have no involvement in providing services nor any interaction with patients. The evaluation will instead rely solely on routinely collected facility-and patient-level records that can be accessed without individual patient consent. Based on our experience in previous studies, we anticipate that each study clinic will have slightly different existing data collection procedures in place, and different provinces may use different versions of standard forms. Study procedures will therefore be adapted on a site-by-site basis before individual patient data collection begins. The National Department of Health will be asked to assure and facilitate access to Tier.Net, DHIS, patient registers and records, and other routinely collected sources of data from the evaluation clinics.

For Objectives 1-5, routine data sources will include Tier.Net data, the National Health Laboratory Service (NHLS) data which contains all laboratory tests done in public-sector clinics, data sets created by entering clinic registers, adherence plans, and patient clinic files into a database. We note that some strengthening of existing data collection procedures will be required at the facilities where the evaluation will take place in order to ensure complete entries into existing clinic registers or patient files, complete and accurate entry of source data onto electronic files, and the use of a consistent clinic-level patient identifier to link patients between data sources (e.g. a register containing a row for each visit and a patient file containing documents pertaining to that patient will each contribute to the evaluation record for that patient). Prior to the start of data collection, we will work with each study clinic to ensure that the registers, stationery and records mandated by the guidelines and the National Health Act are being completed correctly and fully, including new documentation called for by the Adherence Guidelines, such as the individual patient Adherence Plan (AGL SOP Booklet, Annexure 1, p. 66).

For Objective 6, to estimate the overall impact of the package of interventions at each study clinic, we will collect aggregate, clinic-level data on numbers of patients presenting in the most recent 12-month period who would be eligible for the interventions. These data will be similar to the aggregate DHIS indicators routinely reported to the Department of Health and will not pertain to individual human subjects.

Unit cost data for Objective 7 will be collected from invoices and price lists and from other public sector information sources. These data will not pertain to individual human subjects.

To describe the care cascade and treatment adherence for TB, hypertension and diabetes under Objective 8, routine data collection at clinics will need to be strengthened. We and the NDOH, supported by implementing partners, will assist sites that are not already using it to adopt the “Chronic Patient Record” included in the NDOH’s Integrated Chronic Disease Management (ICDM) Manual (Tool 29 on pages 110-11 of the manual, Appendix III)[14]. (Provincial DOH Chronic Patient Records may also be used) This form, if fully completed, will contain most of the data required to achieve Objective 8. Forms will be linked to other patient records, such as the PHC Tick Register, Adherence Plan and NHLS laboratory test reports, as needed to provide the full data set.

## **b. Data Fields to be collected**

Below (Table 2) we summarize the existing data required for the evaluation. A detailed list of data fields follows this summary.

**Table 2. Data required for the evaluation**

| **Objective** | **Data that must be fully captured by routine data collection systems and procedures** |
| --- | --- |
| Fast track ART initiation counseling (Objective 1) | Scheduled and actual visit date information, CD4 counts and dates when results are received, date at which ART eligibility is determined, date of first ARV dispensing, viral loads. |
| Enhanced adherence counseling (Objective 2) | Scheduled and actual visit information, date of eligibility for enhanced adherence counseling, dates of delivery of counseling, viral loads. |
| Adherence clubs (Objective 3) | Club membership, scheduled and actual visit/club participation information, date of eligibility, dates of all medication pick ups, viral loads. |
| Decentralized medication delivery (Objective 4) | Scheduled and actual visit information, date of eligibility, dates of all medication pick ups, viral loads. |
| Early tracing (Objective 5) | Scheduled and actual visit information, date of eligibility, numbers and types of tracing attempts made, results of tracing, viral loads. |
| Overall impact (Objective 6) | Aggregate figures of total numbers of patients presenting and eligible for each intervention (aggregate at clinic level).*  (*Note: as these data do not pertain to individual human subjects, they are not included in the list of fields below.) |
| Cost estimates of interventions (Objective 7) | Unit costs of all resources utilized to implement the interventions.* (*Note: as these data do not pertain to individual human subjects, they are not included in the list of fields below.) |
| TB, diabetes and hypertension (Objective 8) | Records for all patients recently screened for these conditions, all laboratory tests and clinical investigations done relating to these conditions (including blood pressure, HbA1c test and TB test results), scheduled and actual visit information, dates of eligibility, dates of all medication pickups, and outcome data. |

The specific data fields available and required for each cohort will vary slightly (e.g. viral loads will not be available for patients newly initiating treatment under Objective 1). In general, however, we will collect a common set of variables for all patients enrolled Cohorts 1-5, and a different set of variables for patients enrolled in Cohort 8, as listed below.

*Objectives 1-6, all cohorts:*

- Electronic medical record system number (e.g. Tier.Net record number)*
- Clinic file, register, and card numbers (assigned by the clinic)*
- National ID number*
- Name*
- Date of birth*
- Weight and height or BMI
- Sex
- Age in years
- Marital status if recorded
- Employment status if recorded
- Education level if recorded
- Smoking and alcohol use if recorded

*To be used to link electronic and paper-based records pertaining to individual patients. Many clinics use name or initials and date of birth to create a clinic record number.

*Objectives 1-5, HIV cohorts:*

- Date of positive HIV test (if available)
- Dates of all clinic visits (including pre-ART and after ART initiation)
- Primary purpose of all clinic visits (e.g. HIV test, CD4 count, counselling, ARV dispensing, ART monitoring, etc.)
- Dates of next scheduled clinic visits (dates patients are expected to return to clinic)
- Date treatment eligibility determined, if reported
- Date and result of CD4 count indicating treatment eligibility (baseline)
- WHO stage and clinical conditions
- Date and result of TB symptom screen
- Date, type, and result of TB test
- Date TB treatment initiated, if relevant*
- Date and result of cryptococcal meningitis test
- Date cryptococcal meningitis treatment initiated, if relevant*
- Date ART initiated (first dose of medications dispensed)
- First-line regimen prescribed
- Any changes to first line regimens and dates of changes
- Dates and results of all viral load tests
- Dates and results of all other laboratory investigations
- All completed fields in Adherence Plan, if entry into an electronic database is feasible (attached)
- Dates of eligibility for receiving interventions
- Dates of participation in intervention activities or receipt of intervention services (e.g. Adherence Club visits, tracing attempts, counseling sessions)
- Dates interventions received if documented

*These conditions require a delay in ART initiation and are thus important in evaluating Cohort 1.

*Objective 8, TB, hypertension, and diabetes cohort:*

- All fields in the Chronic Patient Record (attached). (Note: These fields may be recorded by the sites in other formats, such as registers or patient files, if the Chronic Patient Record is not yet in use; we will extract them from whatever source documents are available.)
- Dates and results of screening tests
- Dates and results of all laboratory investigations
- Dates of all clinic visits
- Prescribed medications
- Primary purpose of all clinic visits

## **c. Duration of Follow Up**

Study enrollment will begin on the date of determination of eligibility for an intervention (Cohorts 1-5) during the enrolment period. Enrolment for cohort 8 will begin if date of eligibility (screening or diagnosis date) was in the last month for TB and Hypertension patients and within the last 6 months for Diabetes patients.

Passive follow up through medical record review will continue for a minimum of 14 months after the date of enrollment (two additional months beyond twelve months to allow one year outcomes to occur). This will allow all subjects sufficient follow up time to complete each of the primary outcomes designated. Patients enrolled early in the study will have up to 20 months of passive follow-up, assuming a six-month enrollment period. Medical records will be checked for each patient after each clinic visit.

## **d. Data Entry and Storage**

During the enrollment period, which we anticipate to last for approximately six months, patients will be identified and enrolled from TIER.Net (Cohorts 1-5) and site records (TBHD cohort) consecutively until sample size targets are met. “Enrollment” will consist only of reviewing site records to select patients who have become newly eligible for the study in the previous interval; there will be no contact with patients for study purposes.

As explained above, the study will not generate any new data beyond what is called for by existing guidelines and procedures. To allow a valid evaluation, we will make efforts to ensure that the data the clinics are supposed to record are collected and documented completely and promptly. Study staff will visit the sites regularly to obtain electronic data sets, capture paper records and registers on electronic case report forms on dedicated tablets, and review data quality. If needed, study staff will assist the sites to complete all routine forms and records and enter them into a database. Once initial data collection systems are in place, we anticipate visiting each site at least every 10 days to collect data sets, review procedures, and respond to data queries from the previous visit. In order to ensure a secure transmission, the electronic data sets obtained from the clinic will be transferred from the study site via a dedicated secure VPN onto protected drives at the local office.

We anticipate entering all data not captured electronically at the facility onto electronic case report forms on tablets on site, so that paper forms do not need to be removed from the study clinics. Once patient information is captured on the tablet, the information will be immediately sent to a highly secure cloud server and wiped from the tablet. The data will then be downloaded onto secure, protected drives at the local office and at Boston University via a dedicated secure VPN. In some cases (e.g. if there are power failures or other difficulties with using computers on site), it may be necessary to enter data onto paper study forms and then enter them into a database at the local office. These forms will be stored in a locked cabinet at the office, which is itself a secure facility, with access to all study cabinets limited to the study team. All electronic data files will be stored on secure, protected drives at the local office and at Boston University, with access limited to relevant study staff.

All subjects will be assigned a seven-digit, sequential identification number. The first three digits will identify the facility, the fourth digit will indicate the study population/cohort and the remaining three digits will be a consecutive number based on subjects as they are enrolled and will specify the individual subject. The study ID number will be used to identify individual subjects in the study databases and for all data analysis.

For each subject, a national ID number, electronic clinic record number, date of birth, and name will be collected to allow linking of fields extracted from multiple data sources (registers, records, lab reports, etc.). An electronic linking file will be created to link this information to a patient ID number. The linking file will be stored separately from all other data on secure laptops or tablet computers and will be transferred between the head office, where it will be created, and field staff only via the secure cloud server or VPN with access limited to the study team. The linking file will be kept until all data collection is complete and all data has been linked. Once the linking is complete, the linking file will be destroyed. All data collection files will be coded using patient ID numbers and not contain subject identifiers.

MSAcess or a similar software program requiring secure log-in and access by invitation will be used to create an electronic database to manage study data. The database will be managed at the HE2RO Johannesburg office by the study team. On a regular basis, the data will be converted to SAS, STATA or SPSS for cleaning and data analysis. Quality of the data will be assured by comparing a subset of values in the database to the study patient files. Additionally, data files will be reviewed by the study team on a monthly basis and queries returned to the site-level staff for response. All data management and analytic databases will be password protected with access restricted to the members of the study team. Any linking files created for data collection will be destroyed once data entry from source records has been completed and the analytic data set cleaned and closed. A fully de-identified data set will be made available through one or more open access portals when the study is closed.

# DATA ANALYSIS

## **a. Outcomes**

Table 3 lists the primary and secondary outcomes we will measure for each of the objectives. Each primary outcome includes both a short term (S) outcome and a longer term (L) outcome for assessment of the immediate and longer term outcomes of the intervention. Our ability to assess secondary outcomes will depend on data availability and duration of follow up. Note that viral suppression in the current South African ART guidelines is defined as viral load below 400 copies/ml^3^, and this is the threshold that the National Health Laboratory Service will report, and is therefore what we will use.

**Table 3. Evaluation outcomes**

| **Objective** | **Primary Outcome** | **Secondary outcomes** |
| --- | --- | --- |
| Fast track ART initiation counseling (Objective 1) | Proportion of patients who initiate ART within 30 days of becoming ART eligible (S) and the proportion of patients who are alive, in care, and virally suppressed (< 400 copies/ml^3^) within nine months of ART eligibility (L). | Proportion of patients who initiate ART within one week of becoming ART eligible  Demographic and clinical characteristics of patients who do and do not achieve primary outcomes (age, sex, baseline CD4 counts, TB diagnosis, other characteristics as allowed by data). |
| Adherence clubs (Objective 2) | Proportion of patients eligible for participation in an adherence club who receive all medications within the first four months after club eligibility (S) and the proportion virally suppressed (< 400 copies/ml^3^) at twelve months after club eligibility (L). | Proportion of patients consistently participating in club  Demographic and clinical characteristics of patients who do and do not achieve primary outcomes. |
| Decentralized medication delivery (Objective 3) | Proportion of patients eligible for decentralized medication delivery who receive all medications within the first three (S) months after delivery eligibility and viral suppression (< 400 copies/ml^3^) twelve months after delivery eligibility (L). | Proportion of patients consistently receiving medications  Demographic and clinical characteristics of patients who do and do not achieve primary outcomes. |
| Enhanced adherence counseling (Objective 4) | Proportion of patients with an elevated viral load who are alive, retained in care and resuppress their viral load (< 400copies/ml^3^) within three (S) and twelve months (L) of eligibility for enhanced adherence counseling. | Demographic and clinical characteristics of patients who do and do not achieve primary outcomes. |
| Early tracing of patients lost to follow up (Objective 5) | Proportion of patients eligible for early patient tracing who return to care within three (S) and twelve (L) months of eligibility. | Proportion of patients reached by tracers  Number of tracing attempts required; proportion of patients retained in care for at least one additional routine visit after tracing  Demographic and clinical characteristics of patients who do and do not achieve primary outcomes. |
| Overall impact (Objective 6) | Proportion of population in need at each clinic who achieve the short term primary outcomes defined above. | Proportion of population in need at each clinic who achieve the long term primary outcomes defined above. |
| Cost  (Objective 7) | Average cost per patient receiving an adherence intervention | Average cost per primary outcome achieved; average cost per 1000 patients receiving the observed mix of interventions |
| TB, hypertension, and diabetes (Objective 8) | For each condition, proportion of patients eligible for treatment who have 80% visit compliance in the first three months after diagnosis (S) and who achieve disease control at the six-month visit after diagnosis (L) as indicated in “Primary Care 101,” the NDOH’s guide for primary health clinic services[15]. The six-month visit will be represented by the visit closest to six months within the window of 5-8 months after diagnosis. The disease control indicators to be measured, with page references for Primary Care 101, are:  TB: cured or treatment completed indicated as the patient’s status at 6 months (page 57)  Hypertension: blood pressure controlled at 6-month visit, with control defined as < 140/90 (or 120/70–140/80 if diabetes, or <130/80 if CVD, heart failure or kidney disease) (page 74)  Diabetes: random glucose < 8 or HbA1c ≤ 7% at 6 month visit (page 71) | Among patients screened, prevalence of pre-diabetes elevated blood sugar (defined by Primary Care 101 as random glucose between 7.8 and 11) and pre-hypertension elevated blood pressure that require monitoring  Proportion of patients diagnosed with pre-diabetes or pre-hypertension who a) progress to diabetes and hypertension; or b) remain in care and receive appropriate monitoring for their condition over 12 months  Prevalence of diabetes, hypertension and TB among patients screened  Proportion of diagnosed patients initiated on appropriate treatment  Proportion of patients on treatment retained in care at 12 months. Loss to follow up will be defined as being 30 days late for a scheduled appointment.  Demographic and clinical characteristics of patients who do and do not achieve primary outcomes. |

For all outcomes under Objectives 1-5 and 8, retention in care will be defined as (1-% attrition), with attrition calculated as the sum of reported deaths, loss to follow up, and reported transfers to other facilities. Retention will thus be interpreted as “retained in care at facility,” since the outcomes of patients who transfer will not be known. Where national identification numbers are available, the national death registry will be searched to locate unreported deaths. Loss to follow up will be defined as failure to attend the clinic within 90 days of a scheduled appointment, as stated in the Adherence Guidelines (Table 7 page 49).

## **b. Sample Size**

Table 4 shows the sample size that is required to detect meaningful differences for Objectives 1-5 and for estimating precise rates for Objective 8. Each sample size is determined to measure our short term outcome for the objective. For Objectives 1-5, all calculations assume a site-clustered design with the clinic as the cluster; 24 clusters evenly split (randomized) between intervention and comparison groups; a coefficient of variation of 0.1, 80% power; and an alpha of 0.05.

**Table 4. Sample sizes for each objective**

| **Objective** | **Sample Size** | **Rationale** |
| --- | --- | --- |
| Objective 1—Fast Track ART Initiation Counseling | 720 patients | The RapIT study of rapid ART initiation, conducted at a well-managed PHC in Gauteng Province, found that about 60% of ART-eligible patients initiated under standard care within 30 days. Conservatively assuming 60% initiation without the intervention and 75% with the intervention, 30 subjects in each of the 24 clusters for 720 total subjects will be required to detect a difference of 15%. We have increased this by 20% to account for ineligible patients. |
| Objective 2—Adherence Clubs | 576 patients | Data from Themba Lethu Clinic show that about 80% of patients made all of their medication pickups over a three month period. It is anticipated that 24 subjects per clinic for a total of 576 patients will be needed to detect a difference of 15%. We have increased this by 20% to account for ineligible patients. |
| Objective 3—Decentralized Medication Delivery | 576 patients | Data from Themba Lethu Clinic show about 80% of patients made all of their medication pickups over a three month period. It is anticipated that 24 subjects per clinic for a total of 576 patients will be needed to detect a difference of 15%. We have increased this by 20% to account for ineligible patients. |
| Objective 4—Enhanced Adherence Counseling | 1008 patients | Data from KZN indicate that 52% of patients with a detectable viral load re-suppress after one session. It is anticipated that 42 subjects per clinic for a total of 1008 patients will be needed to detect a difference of 15%. We have increased this by 20% to account for ineligible patients. |
| Objective 5—Early tracing of patients lost to follow up | 576 patients | Data from various Right to Care clinics suggest that the proportion of patients who are lost from care who return with no or little intervention is low, between 20-35%. It is anticipated that 24 subjects per clinic for a total of 576 patients will be needed to detect a difference of 15% assuming a baseline of 30% loss to follow up without intervention. We have increased this by 20% to account for ineligible patients. |
| Objective 6—overall impact | 0 patients | No additional subjects will be enrolled for the overall impact analysis, which will rely on data from Cohorts 1-5. |
| Objective 7—Cost | 0 patients | No additional subjects will be enrolled for the cost estimates, which will rely on data from Cohorts 1-5. |
| Objective 8—TB, hypertension, and diabetes | 4800 patients | Because Objective 8 is descriptive in nature, no specific sample size will be targeted. We will instead include up to the first 100 patients per site who are eligible for screening and 100 patients who are recently diagnosed and treated for TB, hypertension and/or diabetes, for a total of 4800. (We note that some patients eligible for the TBHD cohort will also be eligible for Cohorts 1-5, due to co-morbidities with HIV. These patients will be co-enrolled in both cohorts.) |

Total enrollment will thus be 2,880 in cohorts 1-5 and 4,800 in the TBHD cohort, for a total sample size of 6,680.

Due to the size criterion for study site selection, with all clinics included in the evaluation having at least 1,000 current ART patients, we anticipate being able to enroll the target sample sizes for all cohorts during a 6-month enrollment period. Enrollment for Cohorts 4 and 5 and 8 may take longer than this depending on baseline rates of viral suppression and visit compliance at the selected clinics and number of newly screened and diagnosed TBHD patients.

We will monitor the data for Cohorts 1-5 closely and amend the protocol to increase the sample size for these cohorts if needed to ensure adequate analytic power.

## **c. Data Analysis Objectives 1-5**

Our general analytic plan will be the same for each of Cohorts 1-5. We will begin with descriptive analyses of the characteristics of each of the cohorts stratified by intervention/non-intervention (comparison) cohorts. We will also look for differences within the randomized matched pairs. Because the data will be collected as part of a clustered design, the data analysis will need to account for clustering. For each primary outcome described above, we will conduct a crude analysis comparing the proportion of subjects with the outcome in the intervention and comparison arms. Next we will conduct an analysis for each outcome accounting only for clustering using generalized estimating equations (GEE) with an unstructured correlation matrix and clustering by treatment site. In all cases the outcomes are dichotomous and therefore we will calculate relative risks comparing the intervention to the comparison arms using a log link function and a binomial distribution. Next, should any imbalances between treatment groups be detected, we will adjust for those covariates in our GEE model. Finally, we will look for differences in the effects of the strategies by important baseline characteristics (e.g. size of the treatment population, rural vs. urban, province, etc.) using stratified analyses.

We note that because each of the interventions is targeted at a different population with the exception of decentralized medication delivery and adherence clubs, which would not be delivered to the same people at the same time, we can estimate the impact of each intervention separately, as only one of them will be delivered to each cohort within a clinic at any one time. Since some patients may participate in more than one intervention (e.g. fast track initiation followed by tracing), we will conduct a sub-analysis restricted to those who received only one intervention to look for the effects of each intervention by itself.

## **d. Data Analysis Objective 6**

To provide an indicator of the overall (combined) effect of the package of interventions, we will analyze patient retention in care within intervention and comparison groups overall. We will include interaction terms between site and intervention to determine whether different packages of interventions produce different cumulative effects. Then, using available data on the population of patients eligible for each intervention at each site, we will weight the effects of the interventions in proportion to population to produce an estimate of the proportion of population in need expected to benefit from the interventions at each site.

## **e. Data Analysis Objective 7**

For Objective 7, we will estimate the incremental cost to the provider (NDOH) of delivering each intervention per patient receiving the intervention, per patient achieving the primary outcome, and per clinic population eligible for the intervention. We will start with bottom-up costing of the resources utilized per patient served in the intervention clinics and recorded in the data sets collected under Objectives 1-5. Costs to be included are those identified in the cost analysis of the Adherence Guidelines we previously prepared for the NDOH[16]. These will be categorized as incremental or non-incremental to indicate whether they are additional budgetary expenses incurred by NDOH or a reallocation of existing resources. The quantity of each resource used by each enrolled patient will be estimated and then multiplied by the unit cost for that resource. Unit costs will be calculated from clinic invoices, NDOH and NHLS price lists, government salary scales, and other sources of actual costs incurred. We will use a top-down approach to estimate shared costs (e.g. clinic management) where necessary. We will closely follow the methods of the previously prepared cost analysis[16] to provide consistency of estimates. This analysis, in turn, follows the methods used in a number of papers we have published on the costs of HIV care in South Africa[17–19].

Once an average cost per patient receiving the intervention has been estimated, we will then estimate a cost per patient achieving the primary outcome (total cost of the intervention per site/total patients receiving the intervention), which is a preliminary measure of the cost-effectiveness of the interventions and can be compared to information from other published sources. We will also estimate the cost to offer the intervention to the full population in need at that site. The sum of these costs for all five interventions will indicate the cost to NDOH of implementing the minimum package at clinics of various sites. Finally, we will use the results of the evaluation to refine the previously completed cost analysis mentioned above[16], which will assist the NDOH to use the results for budgeting purposes.

If data collected on the TBHD cohort are sufficient to capture all resources utilized by the clinics for TB, hypertension, and diabetes care, we will use the same bottom-up cost methods to estimate the costs of care for these conditions per patient treated.

## **f. Data Analysis Objective 8**

For Objective 8, we will describe retention in each stage of TB, hypertension, or diabetes care over time using simple proportions for each stage in the care cascade, stratified as data allow by clinic, province, sex, age group, and HIV status. Estimates will be presented alongside their corresponding 95% confidence intervals to allow evaluation of whether adequate precision was achieved. We will describe time to events (primary outcome, loss to care) using Kaplan-Meier curves. Finally we will look for predictors of poor retention for each condition using logistic regression and Cox proportional hazards regression.

## **g. Dissemination of Findings**

The primary audience for this evaluation is the South African National Department of Health and its partners, which will use the results to improve, target, and budget for the national implementation of the Adherence Guidelines. Many of the findings, however, will likely be of broader interest in South Africa and other countries, where effective strategies for improving chronic disease medication adherence are eagerly sought. Results of the evaluation will be made as widely available as possible, through journals, websites, and conferences. Only aggregated, stratified data will be presented and it will not be possible to identify any individual patients from any of the data that is presented.

# ETHICAL CONSIDERATIONS

The evaluation will require ethical approval from the Institutional Review Board of Boston University and the University of the Witwatersrand’s Human Ethics Research Committee. In South Africa, national, provincial, and district-level approvals may also be required.

**a. Potential Risks and Protections**

The study team will not collect any biomedical samples specifically for this study nor have any interaction with study subjects. Both biomedical and clinical data for the study will be drawn from existing records that are routinely collected at the study sites as part of routine patient care. We therefore believe that our study poses no physical risks to subjects.

The only risk that we believe is posed by this study is that of loss of confidentiality. We will collect data indicating individuals’ HIV status and other sensitive health information. A high level of stigmatization continues to inhibit the disclosure of HIV status in the study population and may also exist for TB and non-communicable conditions. Since we will only enroll patients eligible for adherence interventions under Objectives 1-5, our data will also reveal poor adherence. A breach of confidentiality, for example through inadvertent loss of a storage device or paper files, would thus pose a risk to subjects.

We will protect against the risk and repercussions of loss of confidentiality in two main ways. First, patient identifiers will be collected and stored separately from all other individual data. Identifiers will be entered on site and stored in encrypted, password protected files, so that no paper records containing identifying information are removed from the sites. As explained above, names, national identification numbers, and other identifying information will be used only for the purposes of linking disparate sources of data for the same patient. As soon as a specific source document has been linked to the patient of interest, data from it will be entered in a record containing the Study ID number only. Analytic data sets will not contain any identifiers, and the linking files containing the identifiers will be destroyed once all linking has been accomplished.

Second, all study data, in electronic or paper format, will be stored in secure locations. Password-protected laptops and tablets used on site will be kept in locked and secure locations when not in use. All data collected on tablets is immediately uploaded to a secure cloud server as soon as data collection for a patient is complete and is not kept on the tablets. Patient data extracted from electronic patient systems is extracted in a password protected double-encrypted format and will be uploaded to a secure server at the HE^2^RO office via a dedicated secure VPN. Any paper files will be transferred to the study office on a regular basis (monthly depending on the remoteness of the sites and data collected) and stored on secure servers and in locked cabinets. Study staff will not be permitted to download de-identified data sets for cleaning or analysis except with the explicit permission of a co-investigator, and data sets will not be stored on individual hard drives when not in use. Upon completion of the study, computer files and any data collection forms containing study data will be retained for five years and then destroyed.

All study staff will be trained in Good Clinical Practice, Research Ethics, and study procedures to ensure that they understand both research confidentiality requirements and study confidentiality procedures. Study investigators will monitor data collection on an ongoing basis. They will report to the BU IRB and the Wits HREC any breaches in confidentiality identified. In the event that a breach in confidentiality does occur, staff will be retrained on human subjects protection and confidentiality if possible or removed from the study if the either the breach is too serious or if the PI feels the staff member cannot be sufficiently retrained. Staff will be made aware of this condition on employment.

**b. Direct Benefits**

There are no direct benefits to study subjects enrolled in this study.

**c. Indirect (Societal) Benefits**

The indirect benefits of this study are expected to be large. The National Department of Health is embarking upon national scale-up of a set of interventions that will ultimately affect hundreds of thousands, if not millions, of patients. Generating early evidence of the expected impact and cost of these interventions for HIV patients and the magnitude and characteristics of the population in need of adherence support among TB, hypertension, and diabetes patients has the potential to make the Adherence Guidelines more effective and less expensive nationwide. If the interventions are found to be effective, the health benefits to patients through improved adherence to and retention in care will be large. Patients will also benefit from the streamlining of service delivery, allowing fewer clinic visits and shorter waiting times. The evaluation will also assist the NDOH to target and tailor its interventions and allocate its resources to the patients at greatest need and/or most likely to benefit and help project budgetary needs in future years. Patients at control facilities will benefit from this streamlined improved service delivery of minimum package interventions as the adherence guidelines are scaled-up and rolled out nationally by the National Department of Health.

Because the indirect benefits of the study are large and the risks to human subjects are minimal, we are confident that the benefits justify the risks.

**d. Informed Consent**

We do not intend to seek informed consent for this study, which, while prospective, is a record review only and poses minimal risk to study subjects. The interventions will be provided by the study clinics as standard care under NDOH’s new Adherence Guidelines, not as part of the study itself. The plan to enrol prospectively, rather than waiting for medical record data to accrue and then collect only retrospective data, results solely from the need for continuous data quality review—prospective enrollment will not impose any additional risks on study subjects. As the interventions being evaluated pertain to adherence and retention in care and many subjects will only be eligible for interventions after being lost to care, moreover, requiring consent would make the study infeasible.

**e. Subject Confidentiality**

As explained above, we will take multiple steps to protect subject confidentiality. These are detailed in the paragraph entitled “Potential Risks and Protections.”

**f. Costs and Payments**

Study subjects will not incur any costs from study participation or receive any payments for it.

# REFERENCES

1. Rosen S, Fox MP (2014) Retention on antiretroviral therapy in South Africa: evidence from a systematic review. Johannesburg: HE²RO Policy Brief Number 8, Health Economics and Epidemiology Research Office.

2. Fox MP, Rosen S (2015) Retention of adult patients on antiretroviral therapy in low- ­and middle- ­income countries: systematic review 2008-2013. *J Acquir Immune Defic Syndr* 69: 98–108.

3. Fox MP, Shearer K, Maskew M, Meyer-Rath G, Clouse K, et al. (2014) Attrition through multiple stages of pre-treatment and ART HIV care in South Africa. *PLoS One* 9: e110252.

4. Clouse K, Pettifor AE, Maskew M, Bassett J, Rie A Van, et al. (2013) Patient retention from HIV diagnosis through one year on antiretroviral therapy at a primary health care clinic in Johannesburg, South Africa. *J Acquir Immune Defic Syndr* 62: 39–46.

5. National Department of Health (2013) Health Indicators Update- Antiretroviral Indicators 2013. National Department of Health, Pretoria.

6. World Health Organization (2014) Global Tuberculosis Report 2014. World Health Organization, Geneva.

7. World Bank. Evaluation of interventions to increase the proportion of people living with HIV who are diagnosed, initiated on, adhering to and retained in HIV treatment and care in South Africa. Formative Qualitative Research: Phase 1 Report. The World Bank, Washington DC.

8. Chaiyachati KH, Ogbuoji O, Price M, Suthar AB, Negussie EK, et al. (2014) Interventions to improve adherence to antiretroviral therapy: a rapid systematic review. *AIDS* 28 Suppl 2: S187–204.

9. Govindasamy D, Meghij J, Negussi EK, Baggaley RC, Ford N, et al. (2014) Interventions to improve or facilitate linkage to or retention in pre-ART (HIV) care and initiation of ART in low- and middle-income settings: a systematic review. *J Int AIDS Soc* 17: 19032.

10. National Department of Health (2014) National Consolidated Guidelines for the Prevention of Mother-to-Child Transmission of HIV (PMTCT) and the Management of HIV in Children, Adolescents and Adults. National Department of Health, Pretoria.

11. National Department of Health (2015) National Adherence Guidelines for Chronic Diseases (HIV, TB and NCDs), Version of November 2015. National Department of Health, Pretoria.

12. Médecins Sans Frontières Khayelitsha (2015) ART/TB/PMTCT initiation patient education and counselling model report and toolkit. Medecins Sans Frontieres, Cape Town.

13. Medicins Sans Frontieres (2014) ART adherence club report and toolkit. Medecins Sans Frontieres, Cape Town.

14. National Department of Health (undated) Integrated Chronic Disease Management Manual. National Department of Health, Pretoria.

15. University of Cape Town Lung Institute (2013) Primary Care 101 Guideline 2013/14. National Department of Health, Pretoria.

16. Long L, Rosen S, Chiu C, Batiancila R (2015) Estimate of implementation rollout costs for the minimum package for HIV tuberculosis, hypertension, and diabetes, version of 14 April 2015. Health Economics and Epidemiology Research Office, Johannesburg.

17. Meyer-Rath G, Brennan AT, Long L, Ndibongo B, Technau K, et al. (2013) Cost and outcomes of paediatric antiretroviral treatment in South Africa. *AIDS* 27: 243–250.

18. Rosen S, Long L, Sanne I (2008) The outcomes and outpatient costs of different models of antiretroviral treatment delivery in South Africa. *Trop Med Int Heal* 13: 1005–1015.

19. Long L, Brennan A, Fox MP, Ndibongo B, Jaffray I, et al. (2011) Treatment outcomes and cost-effectiveness of shifting management of stable ART patients to nurses in South Africa: an observational cohort. *PLoS Med* 8: e1001055.
